# Role of Incentives in the Use of Blockchain-Based Platforms for Sharing Sensitive Health Data: Experimental Study

**DOI:** 10.2196/41805

**Published:** 2023-08-18

**Authors:** Pouyan Esmaeilzadeh, Tala Mirzaei

**Affiliations:** 1 Department of Information Systems and Business Analytics Florida International University Miami, FL United States

**Keywords:** blockchain technology, data sharing, health data, clinical research, incentive mechanisms

## Abstract

**Background:**

Blockchain is an emerging technology that enables secure and decentralized approaches to reduce technical risks and governance challenges associated with sharing data. Although blockchain-based solutions have been suggested for sharing health information, it is still unclear whether a suitable incentive mechanism (intrinsic or extrinsic) can be identified to encourage individuals to share their sensitive data for research purposes.

**Objective:**

This study aimed to investigate how important extrinsic incentives are and what type of incentive is the best option in blockchain-based platforms designed for sharing sensitive health information.

**Methods:**

In this study, we conducted 3 experiments with 493 individuals to investigate the role of extrinsic incentives (ie, cryptocurrency, money, and recognition) in data sharing with research organizations.

**Results:**

The findings highlight that offering different incentives is insufficient to encourage individuals to use blockchain technology or to change their perceptions about the technology’s premise for sharing sensitive health data. The results demonstrate that individuals still attribute serious risks to blockchain-based platforms. Privacy and security concerns, trust issues, lack of knowledge about the technology, lack of public acceptance, and lack of regulations are reported as top risks. In terms of attracting people to use blockchain-based platforms for data sharing in health care, we show that the effects of extrinsic motivations (cryptoincentives, money, and status) are significantly overshadowed by inhibitors to technology use.

**Conclusions:**

We suggest that before emphasizing the use of various types of extrinsic incentives, the users must be educated about the capabilities and benefits offered by this technology. Thus, an essential first step for shifting from an institution-based data exchange to a patient-centric data exchange (using blockchain) is addressing technology inhibitors to promote patient-driven data access control. This study shows that extrinsic incentives alone are inadequate to change users’ perceptions, increase their trust, or encourage them to use technology for sharing health data.

## Introduction

### Existing Health Information Exchange and Possible Issues

Health data sharing involves different stakeholders, such as data owners, data users, and regulators. Sharing health data can yield several benefits, including improving care coordination, care quality, and patient safety while reducing mortality rates, medical errors, and health care costs [1]. Sharing health data can contribute to the development of health information exchange (HIE) databases that can be used for various reasons, such as health care (diagnoses and treatments) or clinical research purposes. Of the influential factors, privacy concerns and risks of a data breach have been highlighted as significant barriers to data sharing in the United States [2]. Patients’ concerns about information privacy and security can lead to incomplete information in HIE. Patients do not participate in data-sharing efforts because of concerns associated with information integrity and confidentiality [3]. Incomplete information indicates that HIE systems do not integrate all essential data sources from patients, perhaps because of privacy and security risks. Even patients with privacy and security concerns may overlook data-sharing benefits and their impact on population health. In turn, they may not consent to disclose their data to different providers [4]. Owing to privacy and security concerns, some providers also report legal concerns about sharing patient health information and may choose not to participate in HIE networks [5].

In general, privacy refers to the ability of individuals to keep their personal information and activities hidden or protected from others (eg, companies, groups, or people). It is the right to control how one’s personal information is collected, used, shared, and disclosed [6]. Information privacy denotes individuals’ control over their personal information, including how it is gathered, processed, transferred, and stored by others. In the context of health care, it is the ability of individuals to keep their personal health information (PHI) private and to determine how others use or analyze such information. There are several concerns related to information privacy that individuals may have [7], including (1) data breaches (personal information may be stolen or exposed), (2) collection and use of personal data (organizations collect large amounts of personal data about individuals), (3) lack of transparency (how individuals’ personal data are being collected and used), (4) surveillance (monitoring of web-based activities), (5) discrimination (discriminating against certain individuals or groups), and (6) privacy policies (being difficult to understand, too long, or containing complex legal language).

Security, in contrast, refers to the measures taken to protect individuals from potential harm, such as theft, damage, or unauthorized access to information. Information security protects information and information systems from unauthorized access, use, disclosure, disruption, modification, or destruction [8]. It involves using various measures, including technical, physical, and administrative controls, to ensure information confidentiality, integrity, and availability [9]. Information security can be described in terms of several dimensions, including (1) confidentiality (protecting information from unauthorized disclosure), (2) integrity (protecting information from unauthorized modification), (3) availability (information is accessible to authorized users when needed), (4) nonrepudiation (proving that a particular user has performed a particular action or transaction), (5) authentication (verifying the identity of a user or system entity), and (6) authorization (granting or denying access to certain data). In digital data sharing, security measures may include password policies, 2-factor authentication, encryption, firewalls, access controls, antivirus software, and other safeguards (such as data backup and recovery and employee training) designed to prevent unauthorized access or misuse of sensitive information [10].

Seamless sharing of PHI is a demanding project in the health care system that is considered highly fragmented in the United States [11]. Fragmented health care services may challenge how health-related data are exchanged among health care providers because different health care providers use various exchange mechanisms. In the traditional exchange mechanism, health data are carried by patients using folders, paper, or files on a CD and can be shared with other health care providers through fax, paper mailing, or phone calls [12]. In the second mechanism (ie, direct exchange), a physician directly sends patients’ encrypted electronic medical records to the clinical inbox of a trusted recipient (eg, another provider), mainly through a secure email protocol [13]. The third model is mostly used for research purposes by developing a central data repository that enables secondary uses of aggregated clinical records for quality measurement, disease registries, and public health analysis [14]. In the last model, health care providers develop patient portals to facilitate prescription renewal and appointment requests and share full or part of medical reports with patients to access and view their PHI [15].

Thus, so far, there is no standard data model, data documentation process, and data transfer mechanism in the United States [16]. The main issues with the current exchange mechanisms can be categorized into 4 groups. The first challenge is that mainstream sharing models are mainly centralized and controlled by a health care organization, and they define a minor role for patients in the sharing process [17]. The second concern is that owing to low visibility into the security and transparency of sharing mechanisms, most individuals are less likely to trust the sharing procedures and technologies [18]. The third reason is that the current models do not openly delineate data ownership, and it is not clear who is the owner of clinical data. The last issue is that there is currently little incentive for individuals (patients) to share their health-related data with health care providers.

### Blockchain in Health Care

Previous studies have suggested that blockchain technology could be a promising platform to facilitate information sharing among stakeholders with different interests [19]. Blockchain-based platforms can be used as a technological solution to foster trustworthy relationships between different business entities [20]. According to Lu [21], blockchain solutions can improve the authenticity, security, and confidentiality of information management using the ledger, encryption, and distributed network. By removing intermediaries, blockchain enables data ownership and gives users more control over their data [22]. Data aggregation for research purposes also requires the deidentification of individuals to minimize the risks of privacy violations. Decentralized systems built on blockchain technology can provide pseudonymity for participants participating in the network [23]. Moreover, recent studies indicate that individuals familiar with blockchain-based services are more likely to recognize potential benefits and use the systems [24]. Users who are aware of blockchain characteristics are more receptive to platforms designed based on this technology (eg, blockchain-based HIE). Therefore, these features motivate using blockchain technology in information-sharing efforts in various contexts, such as health care.

### Incentive Mechanisms for Sharing Data

Studies in the health care industry indicate that lack of incentives is one of the most important barriers to information sharing. For instance, Vis et al [25] reported that lack of financial support could negatively influence individuals’ intention to share their genomic data. Regarding sharing clinical data for research purposes, using well-developed systems in web-based health communities positively encourages individuals to engage in sharing health data with others [26]. The ultimate goal of ecosystems for HIE systems is to ensure that patient-level data are securely and efficiently shared nationwide in the United States. Patient-centered health care systems are increasingly willing to give more responsibility to patients in the process of data sharing. This implies that in decentralized systems, patients can control who will access their health data, realize the purpose of data sharing, and recognize the portion of medical data that will be shared in the network. However, such HIE ecosystems are yet to address the requirements in case of transferring data for research purposes. Thus, without a thorough understanding of the decentralized incentivized systems in HIE networks, it is difficult to guarantee that a patient is willing to share their sensitive health information with researchers and health care companies.

Blockchain technology can be used to remove the inefficiencies, costs, and risks associated with traditional data sharing in health care. In the health care industry, researchers increasingly recognize the importance of sharing patients’ data for clinical research. Several private companies have already offered blockchain-based data-sharing platforms [27]. However, the issue with these platforms is finding the appropriate and meaningful incentive mechanism to use the promise of data sharing, relying on the decentralized system for data storage and management.

### Research Questions

The unique features of blockchain, such as decentralization, patient empowerment, increased control and ownership, transparent authorization process, and the use of smart contracts, create a scenario where individuals play a more critical role in the data-sharing process. This makes the role and importance of extrinsic incentives different in blockchain-based HIE systems compared with centralized HIE systems. Previous studies have emphasized that incentives can play a crucial role in blockchain platforms [28]. Incentives can contribute to the blockchain economy by playing the role of digital processes in peer-to-peer (P2P) exchanges that facilitate the value creation of blockchain-based digital goods. The backbone of blockchain that underscores decentralizing decision rights, consensus governance, and technical accountability highlights the importance of incentive alignment. Unless incentives are appropriately aligned, the blockchain nodes will not contribute to its designed premises (such as exchanging health information). Improper incentive options endanger the entire blockchain’s integrity and impede system development, maintenance, and use. A blockchain with aligned incentives motivates users to choose actions that match the goals of the system’s design (eg, data sharing). Although incentives are a vital factor in provoking desirable behavior by those using blockchain-based systems, ensuring that the underlying blockchain infrastructure functions effectively is also important. However, it remains challenging to design effective and appropriate incentives that encourage secure and collaborative data sharing in blockchain-based networks [29]. Thus, creating and applying a proper extrinsic incentive mechanism (offering cryptocurrency, money, or recognition) can strongly affect users’ blockchain adoption behaviors and, ultimately, increase network effects. Therefore, the main research questions that this study aimed to address are as follows:

1. What type of extrinsic incentive option is more significant in motivating users to share their sensitive health information through blockchain?
2. Does familiarity with blockchain technology influence individuals’ perceptions of blockchain-based platforms designed for data sharing?

### Significance of the Study

Several studies have suggested the possibility of using blockchain-based solutions for sharing health data [30]. However, little is known about how individuals perceive and evaluate alternative incentive mechanisms used to encourage people to disclose PHI for clinical research purposes. Thus, this study’s main objective was to shed more light on public perspectives (benefits, concerns, and risks) associated with 3 extrinsic incentive systems (ie, cryptocurrency, money, and recognition) that could be leveraged in blockchain-based platforms designed for sharing health information with researchers. Moreover, we measured individuals’ perceptions of the 3 alternatives using 9 outcome variables. As this study was a field experiment, we did not develop or test a research model. By clearly defining the problem, this study aimed to enhance comprehension and gain fresh insights into the factors influencing individuals’ participation in data-sharing projects with diverse incentive mechanisms. The results can contribute to knowledge by offering how technological means and extrinsic incentives can make health information sharing in blockchain-based platforms attractive. Our study can provide implications for the incentive theory of motivation by exploring how blockchain network users should be compensated for sharing their sensitive health information. The findings may help research organizations craft the right incentive programs that are more likely to encourage individuals to share their sensitive health information. The results can also be important to system designers because the entire blockchain integrity will be threatened without aligned incentive mechanisms, and network effects will be lost. The findings can suggest practical contributions by investigating how blockchain developers may predict the needs and incentives of network participants. Finally, this study can help research companies using blockchain technology to realize how incentives can be best provided to users to develop and maintain blockchain-based platforms.

### Literature Review

#### Blockchain Technology

There is an increasingly growing interest among researchers to examine the potential of blockchain in various industries. As a disruptive technology, blockchain provides a decentralized network and a distributed digital ledger for all the participating stakeholders [31]. No central authority or server controls the blockchain; thus, blockchain-based platforms facilitate P2*P* value transfers of all types (such as financial assets or data) [24]. Blockchain uses a distributed database, decentralized and immutable mechanism, and secure cryptographic algorithms that control each block in the chain [32]. The use of blockchain is no longer limited to the financial sector. Several studies report use cases of blockchain-enabled solutions in different industries, such as supply chain management [33], the energy sector [34], and health care [35]. Although numerous potential blockchain applications have been suggested across industries, the widespread implementation of blockchain-based systems remains limited in practice [36].

One practical use of blockchain is sharing health information [37]. Previous studies have presented the potential advantages of blockchain technology in response to traditional risks associated with conventional information exchange models [38]. For instance, in the context of HIE, a permissioned blockchain-based network is suggested as a more secure option to enable the electronic exchange of clinical data with providers [39]. As medical records are considered sensitive data, legal, consent, and privacy concerns are the top challenges that individuals may encounter in sharing their health information [40]. Existing data-sharing models mainly use central data management mechanisms, making data ownership and controlled access more complicated for individuals. Centralized applications cannot allow multiple stakeholders to actively participate in data-sharing governance. Furthermore, because of the nonautomated consent mechanisms and data access management, the custody and administration of data sharing using traditional HIE are complicated [41].

Therefore, decentralized platforms using encrypted databases are considered an effective alternative enabling independent stakeholders to supervise data contribution and access [42]. As there is neither a central administration nor a third party, trust will be placed in the network and distributed ledger to collect, store, and validate data sharing among data contributors [43]. Thus, previous studies offer blockchain-based platforms for health data transmission between stakeholders such as patients, providers, hospitals, and research organizations [44]. Decentralized networks of distributed nodes are deemed useful to reduce the inefficiency, costs, trust, and security risks of using central data sets across different boundaries. Data transmission through blockchain platforms can enable data contributors to maintain an autonomous and ongoing control of their own data [45]. In these P2P platforms, each node comprises network participants (eg, patients) who help validate transactions and store identical copies of all data-sharing records.

#### Incentives for Data Sharing

Previous studies mentioned that adding incentive mechanisms to distributed networks can increase users’ willingness to participate in data sharing [46]. Blockchain-mediated platforms give rewards to miners who validate transactions [47]. In a private data-sharing network, data contributors can store their health data in a block. Then, they can allow other stakeholders (such as researchers) to seek and buy the data. Thus, the network can help mediate the storage and selling of health data, and individuals remain in control of their PHI [48]. Several blockchain-based networks are in place to incentivize patients to share their health data for research and health care purposes. The existing incentive systems for data sharing can be categorized into 3 groups: cryptocurrency, money, and recognition. Literature indicates that in blockchain-based data-sharing platforms, data owners do not only enjoy more transparency and protection of data but they will also be incentivized with digital tokens [49]. A recent study proposed a blockchain-based system for sharing health record electronically, which rewards the data owner with cryptocurrency for each search [50]. Makhdoom et al [51] designed a blockchain-based framework for secure data sharing in smart cities, which rewards the users with a local digital token for sharing their data with the stakeholders or third parties. Ozercan et al [52] explained how blockchain can be used in genomics by storing sensitive files and rewarding users with cryptocurrency. Some networks use a community-owned database to reward individuals who share their DNA data, lifestyle data, and other health information with scientists through a secure platform. In these networks, individuals are not the subjects of research but they are partners in discovering new treatments. For example, Luna Coin [53] incentivizes data owners to share their health information with genomic researchers to receive Luna Coins, which can be exchanged with other cryptocurrencies (such as Bitcoin and Ethereum). In this decentralized platform, one copy of individuals’ health data exists; they can control its inclusion in the network, and they can release consent to how it is used in research. All the health data are deidentified and stored in the network, and if individuals do not want to contribute to health research anymore, they can remove their health data from the platform.

In the second category, individuals’ PHI data are deidentified, accumulated, and encrypted to ensure privacy and security. Researchers pay to research the aggregated data, and patients earn money for participation or data sharing for clinical research. Patients remain in complete control of their data and are given the ability to acquire a copy of their health records. Psychological studies have highlighted how the concept of money changes personal and interpersonal behaviors [54]. Gabisch and Milne [55] described that offering money can be a good way to increase data disclosure because a monetary value can indicate the profit extracted from the data. Previous studies also examine how offering monetary compensation may affect individuals’ willingness to share their private information with a data broker [56]. An experimental study reported that offering money can increase both the reported willingness and the actual disclosure of personal information [57]. In this model, individuals (data contributors) will control who accesses it and will also have the opportunity to share it with scientists for financial incentives. Data contributors can monetize their health data by anonymously offering controlled access to their PHI to pharmaceutical and research companies. For instance, Shivom (a blockchain that connects patients, genomic donors, medical experts, and the pharmaceutical industry into a single ecosystem) rewards patients for sharing their genomic data on the blockchain by directly selling access rights to third-party institutions. Genome data donors own their data and access rights. This decentralized community offers unique incentives ranging from token rewards to discounted reports and genetic counseling sessions via its partners and affiliates.

The third category gives patients an immutable log of their medical history. When individuals share their health data on these decentralized platforms, they do not only join a community of health advocates that directly advance medicine but also share the value created from their health information. By joining the blockchain-mediated platforms, patients become members of a community, embarking on a journey to drive medical breakthroughs. These communities reward data contributors with recognition and coauthorship. Previous studies have suggested that individuals can use patient-centered HIE platforms enabling data sharing for research purposes in exchange for societal benefits, such as receiving recognition through contributing to national public health [58]. Existing literature implies that blockchain technology can support building up incentives for data owners to share their data in exchange for credits encoded in smart contracts [59]. Data donors will receive credits by providing benefits to society, science, and research communities. These platforms incentivize individuals to share data by offering academic credits and recognition points. For example, the more individuals share genomic data, the more points (stars) they will receive. These points differentiate them in the network by recognizing them as individuals who care about public health and the community’s welfare. Thus, each individual who gives permission to researchers will receive shares in the form of authorship credit. The authorship credits will be integrated into the blockchain-based platforms and shared with others [60]. For example, the holders of the authorship credits will receive recognition for sharing their health data that could be used for clinical research purposes to improve public health quality, reduce health risk factors, and discover the best health care practices [61]. Through partnerships with disease foundations and academic institutions, data contributors will assist in health discoveries and help drive medical innovation for the greater good of humanity.

#### Theoretical Background and Hypotheses Development

Although information sharing has many benefits in health care, one of the main problems that should be solved is the unwillingness to share owing to a lack of incentives [46]. Individuals participate in information sharing as long as the benefits surpass the current and future risks of disclosure. The incentive theory explains that reward learning and motivation concepts help guide goal-directed behavior [62]. It suggests that a reward is offered to the user if the expected behavior is enacted. Thus, presenting a reward can strengthen a given behavior (data disclosure). However, disclosures under no-reward contexts are mainly subject to the intrinsic value expectations of individuals.

Drawing from motivation theories, several studies of blockchain-based services suggest that extrinsic incentives positively affect users’ data-sharing efforts [28]. Studies on intrinsic motivation in decentralized networks indicate that personal growth, skill development, and fun are considered the main intrinsic motivation of users [63]. Generally, individuals who are intrinsically motivated are more likely to perceive the meaningfulness of a particular job. However, in the blockchain context, many users may expect to receive extrinsic incentives (such as digital tokens) to share their PHI with scientists for research purposes [64]. As mentioned in the *Incentives for Data Sharing* section, blockchain networks extrinsically compensate users for the information they disclose on these platforms. The extrinsic value that they expect to derive from such disclosures is the primary driver in this context. Therefore, personal development, self-achievement, and competence may not be the key motivators for users to share their health data for research purposes in a blockchain-based platform. For instance, in this study, users may not regard completing the task (ie, sharing PHI with research organizations) to demonstrate their competence to gain enjoyment and, consequently, allocate more resources to the task. Moreover, users may not be incentivized to perform data disclosure for research purposes only because the act of performing the task offers them inner benefits (eg, entertainment).

Previous studies mention that external incentives could neutralize the inner needs for autonomy and competency and weaken the significance of intrinsic motivation [65]. In the context of blockchain services for information sharing with researchers, incentive mechanisms (eg, monetary rewards or recognition opportunities) are common ways to attract data owners’ interest and attention [48]. As PHI is shared for research purposes (secondary use of data), providing extrinsic incentives would be more meaningful to increase users’ engagement. In this study, we also argue that the presence of extrinsic incentives may overshadow the impacts of intrinsic motivation.

When external incentives are offered, users rationally evaluate the consequences of their information-sharing behavior and then adjust their plans to obtain the incentives [66]. Thus, external incentives can control users’ participation in information sharing and decrease the power of intrinsic motivation to involve them in the blockchain network. When users interpret that their effort for data-sharing tasks is externally driven, they tend to give more weight to the reward than to meeting their inner needs. Presenting external incentives makes users focus on obtaining more external benefits, thereby reducing the effects of intrinsic motivation [67]. Hence, the self-challenge for completing the information-sharing task is no longer a leading predictor of engaging in the sharing efforts. Furthermore, getting recognition through participating in a research project to improve public health care quality is another essential external incentive in decentralized communities [68]. Some users consider sharing their personal information as a way to gain visibility in the network to receive authorship credits. The desire for recognition incentivizes data owners to focus on external-driven outcomes (receiving societal credits) and overlook the satisfaction of the inner needs. Accordingly, the strength of intrinsic motivation to promote engagement in information sharing would be decreased in blockchain-based platforms when extrinsic incentives are available.

Moreover, to measure individuals’ perceptions of blockchain-based platforms, we used the following variables: perceived privacy concerns, efficiency of data sharing, trust in the data-sharing mechanism, transparency of sharing procedures, control, anonymity, data ownership, and willingness to use. First, we included these variables to highlight the main barriers and facilitators of data sharing in health care indicated by previous research. Second, we selected these factors to examine the impacts of 3 extrinsic incentives on data-sharing perceptions in the blockchain context. Several studies have reported that privacy concern is the most critical barrier to disclosing health information [69]. Perceived trust in data-sharing platforms (such as HIE systems) and transparency of data exchange procedures are also considered significant facilitators of health data disclosure [70].

Existing literature proposes that providing ownership rights of health data, sharing deidentified health information, and controlling the authorization process of data sharing are key drivers of using blockchain for sharing sensitive health data [71]. Recent studies suggested that incentivizing data holders can facilitate health data disclosure in decentralized networks [48]. Allowing efficient communication between data senders and receivers in blockchain-based platforms is a critical enabler for information sharing [72]. Individuals who are possibly aware of the availability and benefits of blockchain technology develop a new understanding of the potential impact of blockchain-based services for data sharing [73]. Finally, individuals’ (data owners) willingness to participate in data-sharing networks can result in repeated interactions and improve the feasibility of using blockchain technology in health care for data-sharing purposes [74]. Accordingly, we develop 2 hypotheses as follows:

- Hypothesis 1: There is a significant difference between how different types of incentives (ie, cryptocurrency, money, and recognition) influence individuals’ perceptions about blockchain-based data-sharing platforms (ie, privacy concerns, efficiency, trust, transparency, control, anonymity, data ownership, and willingness to use).
- Hypothesis 2: Familiarity with blockchain technology affects individuals’ perceptions of blockchain-based data-sharing platforms (ie, privacy concerns, efficiency, trust, transparency, control, anonymity, data ownership, and willingness to use) across 3 incentives (ie, cryptocurrency, money, and recognition).

## Methods

### Research Approach

Previous studies have suggested that blockchain technology can be a reliable alternative to existing information exchange mechanisms in health care [75]. In this study, we focused on blockchain applications in the health care context because, first, it is discussed that blockchain could help solve some concerns and risks associated with current HIE methods. For instance, concerns about patient roles, ownership, control, rights, consent, security, and privacy could be resolved through blockchain-enabled networks [39]. Second, using incentives for sharing health information has been an attention-driven approach feasible in blockchain-based HIE systems. Health care companies (such as pharmaceutical companies) have different options to integrate incentives into their information-sharing platforms. The need to find and incorporate appropriate incentives into their blockchain-based HIEs to incentivize health information sharing is a strategic decision for health care organizations.

To achieve this study’s objective, we designed a field experiment by administering a web-based survey to examine individuals’ perceptions of incentive mechanisms for data sharing. We designed 3 separate scenarios for the 3 incentives. The 3 scenarios consisted of a general section and a special feature. In the general section, which was repeated in the 3 scenarios, we asked respondents to think of a decentralized platform (blockchain-based technology) that can share their sensitive health information with scientists and researchers in a secured network for clinical research purposes. The company that provides this technology is called Company X-Block. We defined the company’s offering as follows:

1. A private network to mediate the searching, storing, buying, and selling of health data
2. Individuals may choose to allow scientists to search for and buy their data
3. Making the individual in charge of their data, and individuals can consent to how their data are used in research
4. Anyone is free to safely store their data on the companies’ network chain
5. There are no personal identifiers to data, and data will be deidentified for sharing purposes

In the special feature of scenarios, which differentiated the 3 scenarios, we clearly described one of the incentive protocols. Scenario 1 explains cryptocurrency as an incentive, scenario 2 refers to money as an incentive, and scenario 3 describes authorship credits and recognition as a reward for data sharing with researchers. Consistent with the study’s purpose, we used a between-subject design, as respondents in each experiment were exposed to only 1 scenario. It should be mentioned that because there was a chance that some respondents might not be thoroughly familiar with blockchain technology or defined incentives, the 3 scenarios and purpose of the study were clearly explained. In this step, we took 2 actions to ensure that the scenario descriptions and incentive definitions were understandable to the general public. First, we consulted 3 well-published scholars in the domain of blockchain and data-sharing efforts in health care. According to the experts’ suggestions, some terms were modified, and technical jargons were removed to ensure that the definitions were transparent to the public. Thus, we discarded several technical terms that could lead to misunderstanding and confusion. For instance, we removed some terms such as decentralized data repository, distributed nodes, cloud-based data repository, encrypted medical records, cryptography, and secured mechanisms. We also avoided any negative or positive connotations and biases with the definitions of incentive mechanisms to reduce the possibility of influencing participants’ perceptions.

Second, the face validity test was conducted with 5 Doctor of Philosophy students (3 in health care and 2 in information systems) to examine whether the survey questions’ simplicity, readability, format, and wording were satisfactory. The face validity check focused on the details of the 3 scenarios and survey items themselves and did not ask participants to rank or respond to the items. A prerequisite for participation in the face validity check was that the participant was familiar with blockchain technology (through research or practice), ensuring that they understood the study’s context. The participants were asked to examine scenario definitions and items and to comment on the clarity of the questions and incentive mechanisms. The main reason for the face validity check was to ensure this study would not be highly technical to the public. To identify technical and unclear definitions and items, we asked the participants to flag items with confusing or vague wording. The definitions and questions were all reported as acceptable, except for a few terms that were recognized as too ambiguous or worded unclearly. The authors also suggested specific changes to certain items or definitions. We then discussed the flagged items, reworded the vague words, and confirmed them with the students to ensure their understandability. The detailed scenarios are provided in Table 1. We have also provided the exact scenarios used in the study in Multimedia Appendix 1.

**Table 1:** Scenario descriptions.

Scenario number	Type of incentive	Description
1	Cryptocurrency	They incentivize individuals to share data by offering cryptocurrency (digital currency). Each individual who gives permission to researchers will receive shares in the form of cryptocurrency. The offered cryptocurrency can be exchanged for other cryptocurrencies (such as Bitcoin and Ethereum).
2	Money	They incentivize individuals to share data by offering money (monetary reward). Each individual who gives permission to researchers will receive shares in the form of money.
3	Recognition (authorship credits)	They incentivize individuals to share data by offering academic credits and recognition points (stars) in the blockchain network. Each individual who gives permission to researchers will receive shares in the form of authorship credits. The authorship credits will be integrated into the blockchain-based platforms and shared with others. The holders of the authorship credits will receive recognition for sharing their health data that could be used for clinical research purposes to improve public health care quality, enhance the quality of health care findings, reduce health risk factors, identify the best health care practices, and discover new treatment and care planning.

### Survey Development

Next, respondents were asked to reflect on their opinions and preferences on the 3 scenarios. We used 9 outcome variables to compare the respondents’ perceptions of the incentive mechanisms. The variable’s definitions were mainly adapted from previous studies with minor changes to the instrument to tailor all questions to a data-sharing context. Table 2 presents the definitions of outcome variables. In the last section, respondents were asked to answer a set of questions about their demographics and experience with blockchain technology. The measurement items used in this study are provided in Multimedia Appendix 2.

**Table 2:** Definitions of outcome variables.

Outcome variables	Definition	Study, year
Perceived privacy concern	The extent to which an individual is concerned about whether their health information is safe from potential compromises (such as errors, data collection practices, unauthorized access, or secondary use of data)	Angst and Agarwal [76], 2009
Perceived trust	The degree to which an individual believes that a data-sharing platform is reliable and trustworthy	Hall and McGraw [77], 2014
Perceived transparency	The degree to which the procedure in which sensitive health data are collected, accessed, and used is transparent	Kim et al [78], 2015
Perceived data ownership	The extent to which an individual believes that a data-sharing platform recognizes their ownership rights on their raw health data	Kish and Topol [79], 2015
Perceived anonymity	The extent to which an individual thinks that a data-sharing platform removes personal identifiers from sensitive health data for sharing purposes	Till et al [80], 2017
Perceived incentives	The extent to which an individual believes that a data-sharing platform offers him or her a particular incentive to share health information for clinical research	Roberts et al [81], 2017
Perceived control	The extent to which an individual believes that he or she can control the authorization and consent process of data sharing	Gordon and Catalini [82], 2018
Willingness to use	The extent to which an individual is likely to use a particular data-sharing mechanism to share their personal health information with a research organization	Esmaeilzadeh [58], 2019
Perceived efficiency	The extent to which an individual believes that a data-sharing platform can efficiently connect data holders and data users	Shabani [48], 2019

### Data Collection

Before data collection, we performed a power analysis to identify the study’s appropriate sample size. The study design comprised 3 scenarios that included 9 constructs measured by a total of 45 items. Considering the study design, the results of the power analysis revealed that for a range of medium (0.5) to high (0.8) effect size, with the probability of making a type I error α=.05 and power β=.95, the total minimum sample required ranges from 41 to 105 per experiment. We initially collected a sample of approximately 203 respondents per scenario to reduce possible sampling errors. Data collection was performed in December 2020 using Amazon Mechanical Turk (MTurk). Previous studies have provided evidence that MTurk is a suitable survey tool for collecting individual-level data [83]. According to Behrend et al [84], participants recruited using MTurk are more representative of the US population in terms of age, gender, race, and work experience.

Moreover, data collected through MTurk have been reported to be more reliable than traditional data collection means and meets the standards of social behavior studies [85]. Researchers, as requesters, can use this crowdsourcing website to reach out to potential participants (ie, MTurk workers) in numerous countries to conduct a survey. Several studies have compared MTurk with conventional data collection methods in the health and medical literature. The vast majority of the studies support the use of MTurk for various academic purposes (eg, in health care research) [86]. Existing literature in clinical research highlights that owing to the large number of users, the MTurk population is more representative of the US population at large than other web-based surveys [87]. We limited the respondents’ location to the United States.

We took several measures and manipulation checks to ensure that the respondents understood the scenarios and provided reliable responses. First, to avoid spammers, we screened the MTurk users by setting the minimum prior approval rate to 95%. Second, we conducted a pilot survey, and following the results of the pilot, we designed short experiments with easy and clear questions to keep the respondents attentive. Third, to minimize the likelihood of participation in >1 scenario per respondent, we used a randomizer function to randomly assign respondents to 1 of the 3 scenarios. We encoded a microcode in the survey to prevent individuals from taking each experiment more than once. Fourth, we double-checked all responses using the generated respondent ID and IP addresses to ensure that the respondents were unique between the experiments. Fifth, we asked participants to rate their familiarity with blockchain technology and their experience in using cryptocurrency. In case participants expressed that “they have used or purchased cryptocurrency,” an additional question appeared to identify which cryptocurrency they had used. Sixth, to avoid random responses and haphazard questions, we incorporated captcha questions in the instrument as a reverse Turing test. We flagged and removed incomplete and careless answers and dropped responses that failed the response quality questions. Finally, to ensure that participants carefully read the survey items, we embedded a numerical code in the survey. We asked participants to insert the code into a textbox at the end of the survey. The incentive for participation was a monetary reward (US $0.50). The average completion time for the 3 groups was 6.3 minutes, which implied acceptable responses in terms of the time spent by respondents for each scenario.

Next, we controlled for several variables using propensity score matching across 3 scenarios considering demographic variables such as age, gender, race, level of education, and income to avoid any potential participants’ biases across groups. We also controlled for participants’ familiarity with blockchain technology and ensured no significant differences in familiarity with the technology across participants in 3 scenarios. Table 3 presents the distribution of the demographic characteristics after matching the participants in 3 scenarios. After matching participants in the 3 scenarios, we had 163 participants in scenario 1, a total of 159 participants in scenario 2, and 171 participants in scenario 3.

**Table 3:** Demographic characteristics per scenario after matching participants.

			Number of participants per scenario, n (%)							Total number of participants in all scenarios (n=493), n (%)			*P* value for *χ*^2^ test	
			Cryptocurrency (n=163)		Money (n=159)		Recognition (n=171)							
**Gender**														.30
	Man	92 (32.7)		98 (34.9)		91 (32.4)		281 (100)						
	Woman	71 (33.5)		61 (28.8)		80 (37.7)		212 (100)						
**Age group (years)**														.43
	<20	0 (0)		0 (0)		1 (100)		1 (100)						
	20-29	41 (30.1)		43 (31.6)		52 (38.2)		136 (100)						
	30-39	63 (36.8)		57 (33.3)		51 (29.8)		171 (100)						
	40-49	34 (30.4)		33 (29.5)		45 (40.2)		112 (100)						
	50-59	18 (37.5)		19 (39.6)		11 (22.9)		48 (100)						
	≥60	7 (28)		7 (28)		11 (44)		25 (100)						
**Income (US $)**														.75
	<25,000	19 (35.2)		18 (33.3)		17 (31.5)		54 (100)						
	25,000-49,999	51 (34.2)		45 (30.2)		53 (35.6)		149 (100)						
	50,000-74,999	40 (29.2)		47 (34.3)		50 (36.5)		137 (100)						
	75,000-99,999	28 (29.8)		32 (34)		34 (36.2)		94 (100)						
	100,000-150,000	22 (44.9)		12 (24.5)		15 (30.6)		49 (100)						
	>150,000	3 (30)		5 (50)		2 (20)		10 (100)						
**Education**														.14
	Less than high school	1 (33.3)		1 (33.3)		1 (33.3)		3 (100)						
	High school graduate	10 (30.3)		10 (30.3)		13 (39.4)		33 (100)						
	Some college	21 (43.8)		8 (16.7)		19 (39.6)		48 (100)						
	2-Year degree	11 (42.3)		11 (42.3)		4 (15.4)		26 (100)						
	Bachelor’s degree	93 (32.6)		88 (30.9)		104 (36.5)		285 (100)						
	Master’s degree	27 (27.6)		41 (41.8)		30 (30.6)		98 (100)						
**Employment**														.08
	Employed full time	132 (31.1)		147 (34.6)		146 (34.4)		425 (100)						
	Employed part time	18 (40)		10 (22.2)		17 (37.8)		45 (100)						
	Unemployed	10 (55.6)		2 (11.1)		6 (33.3)		18 (100)						
	Retired	3 (60)		0 (0)		2 (40)		5 (100)						
**Race**														.72
	African American or Black	14 (42.4)		9 (27.3)		10 (30.3)		33 (100)						
	Asian	14 (40)		8 (22.9)		13 (37.1)		35 (100)						
	Hispanic	7 (30.4)		7 (30.4)		9 (39.1)		23 (100)						
	White	128 (31.6)		138 (34.1)		139 (34.3)		405 (100)						

### Ethics Approval

The institutional review board of Florida International University reviewed and approved the study (approval number 109964).

### Informed Consent

According to the institutional review board approval, written informed consent to participate in the study was obtained from all participants.

## Results

### Overview

We used SPSS Statistics (version 26.0; IBM Corp) to analyze the data. Table 4 shows the correlations among the variables used in this study.

We calculated unweighted sum scores of the items for each variable to create the aggregates of outcome variables. Table 5 shows the summary statistics, including mean score, SE, CIs, number of items, and maximum score per construct across the 3 scenarios.

**Table 4:** Correlation analysis among variables.

Variables	Efficiency	Trust	Transparency	Control	Incentives	Anonymity	Privacy	Data ownership	Willingness
Efficiency	1	0.74	0.71	0.62	0.62	0.67	0.14	0.54	0.55
Trust	0.74	1	0.76	0.61	0.65	0.68	0.07	0.58	0.68
Transparency	0.71	0.76	1	0.73	0.63	0.72	0.11	0.6	0.58
Control	0.62	0.61	0.73	1	0.67	0.74	0.14	0.66	0.61
Incentives	0.62	0.65	0.63	0.67	1	0.74	0.07	0.61	0.74
Anonymity	0.67	0.68	0.72	0.74	0.74	1	0.12	0.69	0.67
Privacy	0.14	0.07	0.11	0.14	0.07	0.12	1	0.22	0.07
Data ownership	0.54	0.58	0.6	0.66	0.61	0.69	0.22	1	0.68
Willingness	0.55	0.68	0.58	0.61	0.74	0.67	0.07	0.68	1

**Table 5:** Summary statistics.

Variables		Sample size	Score, mean (SD; 95% CI)	Values, SE	Range
**Willingness**					
	Cryptocurrency	163	17.94 (4.873; 17.18-18.69)	0.382	5-25
	Money	159	18.97 (3.445; 18.43-19.51)	0.273	7-25
	Recognition	171	18.09 (4.601; 17.40-18.79)	0.352	5-25
**Data ownership**					
	Cryptocurrency	163	18.18 (4.241; 17.52-18.83)	0.332	5-25
	Money	159	18.83 (3.077; 18.35-19.31)	0.244	8-25
	Recognition	171	18.59 (3.727; 18.03-19.15)	0.285	8-25
**Privacy**					
	Cryptocurrency	163	20.02 (5.916; 19.10-20.93)	0.463	6-30
	Money	159	21.75 (4.507; 21.04-22.45)	0.357	10-30
	Recognition	171	21.70 (5.202; 20.91-22.48)	0.398	7-30
**Anonymity**					
	Cryptocurrency	163	19.00 (4.279; 18.34-19.66)	0.335	5-25
	Money	159	19.44 (3.207; 18.94-19.94)	0.254	7-25
	Recognition	171	18.88 (3.796; 18.31-19.46)	0.290	5-25
**Incentives**					
	Cryptocurrency	163	18.37 (4.986; 17.60-19.14)	0.391	5-25
	Money	159	19.13 (3.36; 18.60-19.65)	0.266	9-25
	Recognition	171	18.31 (4.416; 17.64-18.98)	0.338	5-25
**Control**					
	Cryptocurrency	163	18.25 (4.532; 17.55-18.95)	0.355	5-25
	Money	159	19.11 (3.332; 18.59-19.64)	0.264	7-25
	Recognition	171	18.88 (3.869; 18.29-19.46)	0.296	8-25
**Transparency**					
	Cryptocurrency	163	18.33 (4.458; 17.64-19.02)	0.349	5-25
	Money	159	19.29 (3.408; 18.76-19.82)	0.270	6-25
	Recognition	171	18.51 (4.076; 17.9-19.13)	0.312	5-25
**Trust**					
	Cryptocurrency	163	18.41 (4.626; 17.7-19.13)	0.362	5-25
	Money	159	19.03 (3.547; 18.47-19.58)	0.281	5-25
	Recognition	171	18.23 (4.150; 17.6-18.85)	0.317	5-25
**Efficiency**					
	Cryptocurrency	163	22.54 (4.855; 21.79-23.29)	0.380	6-30
	Money	159	23.14 (4.138; 22.49-23.79)	0.328	9-30
	Recognition	171	22.16 (4.788; 21.44-22.88)	0.366	6-30

### Hypothesis 1

To test the first hypothesis, we compared the 3 scenarios using ANOVA to identify whether offering different incentives may influence individuals’ perceptions of blockchain technology. Interestingly, we found no significant differences between the groups in terms of perception of efficiency of data sharing (*P*=.15), trust in data-sharing process (*P*=.19), information transparency (*P*=.08), control over data (*P*=.13), incentive offerings (*P*=.17), anonymity of data sharing (*P*=.38), data ownership (*P*=.28), and willingness to use (*P*=.07). These findings suggest that offering different incentives is insufficient to encourage individuals to use blockchain technology or to change their perceptions about technology’s premise for sharing sensitive health data. This result does not provide evidence to support the first hypothesis. We found significant differences only in terms of privacy concerns (*P*=.003). Participants in scenario 1, in which cryptocurrency was offered as an incentive to use blockchain technology, revealed significantly fewer privacy concerns than the other 2 groups.

### Hypothesis 2

To test the second hypothesis, we divided the participants according to their level of familiarity with blockchain technology within each scenario. We asked respondents to express their prior experience with sharing data using blockchain technology and their familiarity with this technology for research or other purposes. On 5-point Likert-scale items, where 1 represents strongly disagree and 5 represents strongly agree, we considered the participants who selected agree and strongly agree with all 5 items of familiarity as highly familiar with the technology, and the rest of the participants were grouped as less familiar with the technology. The results revealed an interesting pattern. We found significant differences for all proposed constructs between individuals with high and low familiarity. The results indicate that participants who are highly familiar with blockchain technology perceive blockchain technology as significantly more efficient for data sharing (*P*<.001), more trustworthy for sharing data (*P*<.001), and more capable for information transparency (*P*<.001) compared with participants who are less familiar with blockchain technology. Moreover, highly familiar individuals believe that blockchain can protect anonymity for data sharing (*P*<.001) and offer more ownership over data (*P*<.001) compared with less familiar people with blockchain technology. Finally, highly familiar individuals were more willing to use the technology (*P*<.001) compared with participants who were less familiar with blockchain technology.

Surprisingly, those who were familiar with the technology still presented greater privacy concerns than those who were less familiar with it (*P*<.001). This finding provides sufficient evidence to support the second hypothesis. The detailed results of Scheffe post hoc tests provided in Multimedia Appendix 3 reveal the differences between different groups of participants. A consistent pattern can be identified from the results of post hoc analysis. For all constructs except privacy concerns, we observed significantly lower mean scores for participants with less familiarity with the technology than for participants with higher familiarity with the technology, regardless of the incentive offered in each scenario.

For perceived privacy concerns, the findings were somewhat different. When the incentive offered was cryptocurrency, we did not find a significant difference between participants who were highly familiar with blockchain technology and those who were less familiar. Within the group highly familiar with blockchain technology, those who participated in the cryptocurrency scenario indicated significantly fewer concerns over privacy than participants in the scenarios that offered money or recognition as an incentive.

### Suggested Barriers

Furthermore, we asked participants to express their thoughts about the barriers that prevent them from adopting and using blockchain technology to share their sensitive health information. The results reveal that their most frequent concern is privacy and security of data sharing, followed by a lack of trust and lack of knowledge about this technology. Table 6 shows a list of barriers that prevented participants from using blockchain technology.

**Table 6:** Barriers to the adoption and use of blockchain technology for data sharing (N=493).

Adoption and use barriers	Values, n (%)
I do not believe in cryptocurrency	15 (3)
My physicians do not use this technology in their practices	44 (8.9)
I think this technology is complicated	54 (10.9)
Use of this technology may increase my health care costs	59 (11.9)
This technology is still not available in hospitals	69 (14)
Lack of regulations to support this technology	128 (25.9)
Lack of public acceptance	153 (31)
Lack of knowledge and familiarity with this technology	182 (36.9)
Lack of trust in the technology	207 (42)
Privacy and security concerns	242 (49.1)

Regarding privacy concerns, technical security measures in implementing information-sharing platforms are essential in providing an efficient and reliable system [88]. Blockchain-enabled platforms can offer a secure approach to alleviate privacy concerns in electronic health record implementation [89]. Blockchain can enhance security and privacy by limiting participants [90]. For example, previous studies offer a permissioned blockchain that stores patients’ health data securely by giving permits to authorized users to participate in the blockchain [91]. Another example is the implementation of consortium blockchain, where an individual or organization can manage metadata for sharing by controlling the blockchain participants [92]. Therefore, the implementation of a blockchain determines its security. However, in this study, privacy and security concerns were still influential from the perspective of potential users. The first plausible reason is the complexity of different types of blockchain-based HIE (ie, public, private, federated, and hybrid) for individuals. Therefore, potential users may still need training and awareness on terms such as encryption, permissioned platforms, and access-controlled data sharing. The second possible reason is that individuals (who are familiar with blockchain technology) still believe that genomic data are highly sensitive; in turn, sharing such data can raise the likelihood of privacy invasion even when blockchain is leveraged. The third probable reason is that even people familiar with blockchain request more laws and regulations on how other entities and institutions can access sensitive medical data through blockchain HIEs.

We also asked the participants about other possible incentives that may encourage them to use blockchain technology. In total, 27.2% (134/493) the participants expressed that they were willing to use the technology if more evidence of easily secured data-sharing capabilities was offered. In total, 19.1% (94/493) of the participants expressed that they needed to see more evidence of protection and data privacy.

## Discussion

### Principal Findings

Involving patients in data-sharing procedures has been a recent transmission in health care. This health care system has shifted the focus from health care providers to patients to develop a patient-driven care model [93]. This transmission has resulted in new security, privacy, technology, incentives, control, and ownership risks. Sharing health information using other technology (such as query based or direct sharing) is different from using blockchain because of its technological characteristics. Previous studies have discussed how blockchain technology can facilitate transmission by addressing new challenges [82]. For instance, blockchain-based platforms can enable real-time capturing of patient clinical records by facilitating data sharing between data contributors and data users. Blockchain-mediated models can protect privacy and security, provide aggregated data for research purposes, improve patient engagement, enhance consent processes, and provide incentives for data sharing.

In this study, we attempt to investigate whether different types of incentives (ie, cryptocurrency, money, and recognition) can help motivate people to use blockchain-based platforms for sharing their sensitive health information with researchers. Our results highlight that despite the potential of blockchain for data sharing in health care, the public still believes that several barriers will need careful attention. According to our findings, participants in the 3 groups shared the same levels of concerns and risks associated with blockchain. The results indicate that concerns associated with privacy, security, trust, consent process, transparency, and data ownership among the 3 groups have still not been addressed. This is consistent with recent studies showing that blockchain technology is still in its infancy stage, and the public sentiment about using blockchain-based platforms for data sharing is far from favorable [94]. Our results confirm previous studies reporting that offering a reward in exchange for personal information intensifies users’ privacy concerns [95].

Previous studies suggest that using a blockchain architecture that allows owners to control and obtain rewards for sharing their sensitive health records would be a significant enabler of data sharing for clinical research [49]. Our study presents that the high levels of risks and concerns with blockchain technology make it complicated for individuals to consider the lucrative role of incentives for data sharing. Thus, regardless of the incentive type, individuals are less willing to trust and use blockchain platforms to share their health information. This finding does not mean that incentivizing people with digital tokens, recognition, or monetary rewards to share their PHI with interested data seekers is not practical. A trustable, secure, and transparent blockchain-based platform for collecting anonymous patient health data can provide clear accountability of access; maintain complete and updated information; increase data ownership; and keep logs of all accesses, sharing, and uses of medical data. By becoming a member, data contributors will be involved in participatory consent to share their health information for health research. They will enjoy improved transparency and robust protection of health information and also be rewarded with various incentives to share their data with data users. Therefore, they become active participants in a private and decentralized network, benefit from the research data economy (individual incentive), help researchers drive more precise health care, and inform prevention strategies (societal benefits).

We believe that the ineffectiveness of the 3 extrinsic motivations in influencing information disclosure on blockchain platforms does not mean that users already enjoy disclosing information on the blockchain, and they do not require extrinsic incentives to see the activity as attractive. We can interpret that the important effect of incentive mechanisms is overshadowed by the strong concerns with blockchain’s technological underpinning, which are still unsolved from the public perspective. The results determine that individuals still attribute serious risks to blockchain-based platforms regardless of incentive mechanisms. Privacy and security concerns, trust issues, lack of knowledge about the technology, lack of public acceptance, and lack of regulations are reported as top risks. This can imply that if these fundamental concerns and risks are not properly addressed, the rewarding nature of incentivizing people cannot appear prominently to data contributors. The findings demonstrate that although including rewards for data sharing is an exciting trend in blockchain-based platforms, given the unsolved challenges and risks, it remains to be seen whether different types of incentive mechanisms can facilitate data sharing by encouraging patients to share their PHI with researchers. The findings also highlight that familiarity with blockchain technology can affect individuals’ perceptions about sharing data on blockchain-based platforms. This result is consistent with previous studies highlighting the significant role of familiarity with technology in blockchain adoption [73].

Recent developments show that the application of blockchain technology in health care is nascent [96]. Some early solutions show that using blockchain technology can reduce health care data–sharing costs, streamline business processes, and improve access to information dispersed by stakeholders. The use of blockchain technology for sharing data depends on a consensus on the validity of transactions. It also provides the capability to create value for transactions through tokens. This tokenization can be used as an incentive for motivating the adoption and use of this technology. However, our findings reveal that before emphasizing the various types of incentives, the users need to become familiar with and be educated about the capabilities and benefits that this technology can offer. This study shows that incentives alone are inadequate to change users’ perceptions, increase their trust, or encourage them to use the technology.

### Theoretical Contributions and Implications

#### Blockchain Application in HIE: Enablers and Incentives

This study contributes to the understanding of incentives for health information–sharing tasks in the context of blockchain-based platforms. Exploring why data owners would like to participate in blockchain communities to share health information is an important research topic in the health care literature. Our study provides insights into the existing literature by using motivation theory to examine the effects of extrinsic motivation on information-sharing efforts in blockchain networks. Although several studies have proposed using incentive mechanisms for data sharing based on blockchain [46], its relevance to predicting individuals’ engagement in sharing health information with researchers in blockchain-based communities has not been confirmed. Our study investigated the role of incentives (ie, cryptocurrency, money, and recognition) in predicting individuals’ perceptions of blockchain-based data-sharing platforms. Moreover, we delineate the effect of familiarity with blockchain technology on data owners’ perceptions of data-sharing in blockchain-based networks. This study explores the role of motivating data owners through external incentives in blockchain-based communities and encourages scholars to further investigate this essential factor.

We also shed light on the potential of using blockchain technology for sharing sensitive health information. The shift in data-sharing frameworks and moving from institution-based data sharing to patient-driven data access and tracking is an attention-driven health care trend. This trend could essentially change customers’ attitudes and health care organizations’ policies regarding HIE, transparency, and data ownership. Although incentivizing patients to participate in data exchange activities may empower patients and health care organizations to shift from an institution-based data exchange to a patient-centric data exchange, an essential first step is addressing technology inhibitors to promote patient-driven access control and data sharing. Previous studies have proposed that blockchain technology could enable this transition; however, data aggregation, availability, and immutability may pose some challenges to patients [82].

Our study provides implications to the emerging body of literature on the motivation for using blockchain technology in the health care context. Several studies have suggested that blockchain platforms can provide solutions to privacy, user control, and incentive problems in the data-sharing context [59]. In terms of attracting people to use blockchain-based platforms for data sharing in health care, we show that the effects of extrinsic motivations (cryptoincentives, money, and status) are significantly overshadowed by inhibitors to technology use. We highlight that the challenges of blockchain technology remain the main barriers for users to share sensitive health information. One plausible justification is public awareness about blockchain (as a data-sharing method) and the perceived concerns and uncertainty associated with that technology (such as privacy issues and complicated technology). The second reason is the current customer readiness to accept blockchain technology in health care. Although blockchain might have a role in smoothing data-sharing change, our study indicates that several challenges need to be addressed before seeing practical implementations. The third reason is the sensitivity of genomic data regardless of data-sharing mechanisms (eg, blockchain).

Our findings show that even data owners looking for gains remain skeptical when offered extrinsic rewards in exchange for their information in blockchain communities. This is in line with previous studies highlighting that no extrinsic incentive can truly motivate individuals to share their personal data when highly sensitive information is at stake [56]. In addition, existing studies mention that extrinsic incentives could motivate users who are already engaged to put more effort into the task [67]. Our results highlight that individuals are still cautious about participating in blockchain platforms for sharing health information because of several inhibitors. However, the existing external incentives may not play an important role in increasing user engagement in blockchain communities. The presence of extrinsic incentives is a reflection of the value attached to completing the task. However, our study implies that technology inhibitors could mask the realization of value and impair user participation. In the shadow of barriers and inhibitors associated with blockchain technology, external incentives could not provide compensation for the information-sharing effort.

Our results align with those of studies on decentralized networks explaining that users’ motivation intrinsically relates to the nature of the community activities and associated challenges [82]. Engaging in activities that meet a need, fulfill personal values and beliefs, and offer a self-rewarding experience can provide numerous benefits, such as intellectual stimulation, new skills development, and making a positive difference [97]. Our results are inconsistent with a study showing that decentralized platforms use both intrinsic and extrinsic (cryptoincentives and reputation effects) benefits to attract users [98]. However, our results support recent research implying that blockchain platforms for data sharing can be implemented without external incentives [46]. We propose that if the inhibitors (eg, lack of trust, privacy and security concerns, and lack of regulations) are addressed, blockchain platforms can facilitate health information sharing merely by relying on some potential benefits such as improved transparency, ownership, and control.

#### Blockchain Application in HIE: Barriers and Inhibitors

Our study contributes to information systems research by identifying and testing the factors that inhibit the intention to use technology (ie, inhibitors) [99]. These studies explain that we must distinguish inhibitors from the enablers of technology adoption and ask specific questions about “why people choose not to use a technology.” Our study also confirms that the inhibitors of technology use are more than just the opposite of enablers (eg, the opposite of usefulness) and are distinct constructs worthy of research. On the basis of the same line of thought, we suggest that inhibitors would affect technology use beyond that of the enablers themselves. We propose that lack of trust in blockchain technology, privacy concerns, issues with the consent process on the blockchain, lack of transparency of data-sharing procedures, and data ownership concerns are still the main inhibitors that refrain users from participating in blockchain platforms for data disclosure. Consistent with a previous study [100], we suggest that consumer readiness is an inhibitor. In our study, consumer readiness describes the extent to which blockchain technology (as an innovation) can be translated into profits. As public awareness of blockchain applications is still evolving, a lack of customer readiness to use this technology (especially for sharing sensitive health information) can negatively drive users. Our findings delineate that inhibitors have adverse effects on using blockchain for sharing PHI and also on enablers, and these effects vary contingent upon individuals’ familiarity with the technology. Our study calls for more research to examine inhibitors’ effects on predicting user behavioral reactions to different motivators in blockchain-based networks.

### Implications for Practice

#### Familiarity With Blockchain

Our findings can have practical implications for designers of a user incentive model with blockchain to find attractive incentive mechanisms for data owners to share their health information. Our study implies that even individuals familiar with blockchain technology are concerned about their information privacy. Given the distributed nature of blockchain networks, individuals may believe that storing their sensitive medical data on-chain with current technology is unsafe. Further initiatives are required to educate people about alternative approaches to consensus (such as proof of stake) or permissioned blockchain-mediated platforms to mitigate privacy and security concerns. In practice, blockchain-enabled platforms leveraged in health care must hide people’s identities and aggregate deidentified health data for clinical research [101]. Thus, data attributes, such as demographics, are not supposed to be publicly shared. These strategies can be communicated to data contributors using marketing efforts.

Moreover, health care organizations implementing blockchain-led data-sharing mechanisms should use educational programs (such as publishing informative videos) to educate users on a permissioned blockchain. For instance, they need to clearly inform users that data contributors can block private network members from accessing all data, or they can revoke authorization for their health data later. Moreover, research organizations in health care can emphasize using zero-knowledge cryptography to verify data-sharing transactions with a high degree of privacy [102]. Private blockchain networks need to inform users that they must use privacy-related best practices, including the Health Insurance Portability and Accountability Act and General Data Protection Regulation compliance, to ensure data contributors’ privacy, security, and transparency in how health data are shared and used. In addition, if individuals are autonomous digital stewards of their health data stored in blocks, mechanisms to manage their digital data (such as private keys or passwords) will need careful consideration.

#### Key Inhibitors

Previous research identifies security breaches and privacy concerns as essential factors in implementing and using data exchange mechanisms in health care [14]. The findings of this study shed more light on the security and privacy concerns associated with blockchain-based transfer mechanisms. As there is a high public concern related to big data operations and centralized networks [103], the potential of data breaches related to blockchain technology is supposed to be lower than that of alternative models. However, in this study, privacy and security risks were ranked as the top concerns regarding blockchain. This result has a practical implication for decentralized networks to promote consent procedures and security safeguards used in blockchain mechanisms to manage access by clearly presenting what data are collected and how the data can be shared. The transparent consent process will help data owners receive logs of transactions, such as who accesses their medical records, when such data are obtained, and why they are shared. The policy makers and stakeholders of blockchain-based networks need to develop, endorse, and communicate a comprehensive privacy and security framework to provide the details required for the challenging nature of data sharing. One approach to regulating access to valuable data is to integrate access agreements into smart contracts. Smart contracts can guarantee that patient authorization is codified, automated, and executable.

We found that the presence of technology inhibitors and unsolved issues could erode the positive effect of external incentives on blockchain-based information sharing. Thus, blockchain designers and sponsors should be aware of this effect when developing incentive mechanisms to motivate users in the network. Therefore, barriers and inhibitors associated with blockchain technology that could engender meaningfulness, information safety, and privacy should be given more attention and effort before designing extrinsic incentives.

Regarding incentives, it is also worth mentioning that financial motivation (in any form) may not be the only option to encourage individuals to join a blockchain-based platform to share their health data. The main issues associated with monetizing data sharing were (1) identifying whether the incentives will apply to all types of health data; (2) determining data value (ie, how much data contributors will earn); and (3) developing a clear framework to explain the code of ethics, legal issues, and compliance with laws and regulations regarding the use of financial motivations for data sharing. The legal framework needs to articulate compliance with security and privacy regulations, such as Health Insurance Portability and Accountability Act, and financial regulatory requirements, such as Know Your Customer, to verify the identity of clients. The legal framework must describe the terms and conditions of receiving financial incentives. The mechanism of calculating financial rewards should be transparent; for instance, it should be clear how the incentives will be measured if data contributors partially share health data, or how the incentives will be affected by the volume or value of data being shared. Data contributors should be clear about what rewards or contributions they will receive for sharing and how their rewards will be affected if they revoke access later. Users’ inability to commodify specific health information makes it difficult for them to consider the price of different information items.

#### Role of Incentives

The incentive strategies of decentralized platforms should transparently articulate if the value of incentives is a function of data size. An incorrect or ambiguous incentive mechanism could not only motivate people to share data, but it could also confuse both data contributors and data users about the sharing procedure. The results of the open-ended question in our study show that individuals consider easy-to-use data-sharing platforms and robust safeguards to protect data privacy as the top 2 incentives to share their health data via a blockchain-enabled architecture. Thus, blockchains in health care can connect incentives to privacy policies and compliance requirements to improve the widespread use of blockchain technology for data sharing.

In practice, incentives are designed to influence consumers’ behavior by balancing and changing their relative cost and benefit perceptions in their decision-making process for the adoption and use of technology [104]. However, our findings reveal that individuals value the different potential benefits of blockchain technology involving nonmonetary costs. The nonmonetary costs are critically important and are reflected in factors such as trust in technology, privacy risks, security concerns, standardization, and regulations to identify the consumers’ behavior. Therefore, before implementing different incentives, practitioners should address the consumers’ need to trust the technology and learn the functions and associated gains and benefits that blockchain offers. Trade-based incentives in which the incentive pattern is money or other resources of a similar value are not sufficient to adopt blockchain technology. Practitioners should consider offering a combination of trade-based and trust-based incentive schemes, where a successful transaction is reliable and enhances the reputation value of both parties involved in the transaction.

### Limitations and Future Research Direction

First, in this study, we collected data from a sample of respondents from the United States. Care work culture, familiarity with blockchain-based services, and data-sharing platforms are diverse among different countries. Therefore, caution should be exercised when generalizing the results of this study. We recommend that future studies consider participants from other geographical locations, such as countries with limited technology infrastructures required for data sharing. Second, our study used a self-rated sample from a web-based survey to recruit participants digitally. Although we took several measures to provide clear definitions and scenarios, there is still a small chance that some respondents were not completely aware of different data-sharing platforms and may have formed their own perceptions of the IT artifact. Therefore, we suggest that further studies use an alternative method to ensure that participants are knowledgeable about blockchain-based solutions. For instance, future research can recruit the current users of existing blockchain-based platforms designed for sharing health data. Third, owing to the exploratory nature of this study, we did not attempt to develop and test a research model. Future studies can also extend our research by developing a conceptual model using the outcome variables suggested in this study and their possible relationships. Further studies with empirical data are needed to provide quantitative evidence of perceived benefits, risks, and concerns associated with blockchain-based platforms used for data sharing in the health care context. Moreover, the model can be tested for the 3 scenarios separately, and possible variance could be analyzed. Fourth, this study describes key barriers to adopting blockchain-based HIE from participants’ perspectives (such as privacy concerns, lack of trust in blockchain technology, lack of regulations, and lack of knowledge). However, we did not empirically test the relationships between these variables because it is not within the scope of this research. Future studies can examine the causal relationships between these inhibitors, such as the relationship between regulations and trust in blockchain-based HIE. Fifth, identifying legal issues and compliance with using financial motivations for data sharing was not an objective of this study. This could be an interesting research stream for future studies to develop a legal framework used in blockchain-based HIE to comply with financial laws and regulations. Finally, future studies can use a within-subject design in which respondents are exposed to the 3 scenarios simultaneously to compare the popularity of the 3 data-sharing platforms and incentive mechanisms.

### Conclusions

Several health care systems are shifting to patient-centered data-sharing platforms, which aim to provide individuals with elevated empowerment, an increased sense of partnership, and more responsibilities. These patient-focused models encounter many risks and challenges related to patient consent, governance, security, privacy, control management, and patient engagement. Blockchain technology is considered an attractive method of addressing these challenges by creating platforms for secure data-sharing mechanisms. Blockchain can also recognize data ownership rights, improve the authorization process, and enhance the transparency of data-sharing procedures. Blockchain-mediated platforms can facilitate the availability of aggregated eHealth data for research purposes using participatory access control. There has been a growing trend toward using various incentives to encourage data contributors to share their health data in private and decentralized networks. In this study, we examined people’s perceptions of 3 alternative incentives (cryptocurrency, money, and recognition) that blockchain-based platforms could use for data sharing. The results demonstrate that concerns associated with privacy, security, trust, control mechanism, transparency, and data ownership among the 3 groups are still high. Therefore, the heightened perceived risks with blockchain technology still prevent individuals from using blockchain networks to share their PHI with researchers. In the presence of these fundamental concerns and technology inhibitors, incentives cannot significantly convince people to disclose their sensitive health data in exchange for an incentive. Incentive mechanisms could be leveraged in health care only if essential risks are addressed. Our study also indicates the importance of familiarity with the technological underpinning of blockchain. Our experiment shows that the general public is not ready for this transformation because of the lack of knowledge as well as privacy and security concerns in particular. Thus, health care systems have yet to highlight blockchain’s potential benefits and address existing concerns to justify the use of blockchain-mediated platforms for data sharing with the public. More marketing efforts, educational programs, and clear privacy strategies are required to provide a transparent vision of using blockchain for sharing health data for clinical research.

## Appendix

Multimedia Appendix 1 Scenarios used for 3 experiments (ie, cryptocurrency, money, and recognition).

Multimedia Appendix 2 Measurement items.

Multimedia Appendix 3 Results of the Scheffe post hoc analysis.

## Abbreviations

HIE: health information exchange
MTurk: Mechanical Turk
P2P: peer-to-peer
PHI: personal health information
