# Supplementary material for: Role of Incentives in the Use of Blockchain-Based Platforms for Sharing Sensitive Health Data: Experimental Study
Source: J Med Internet Res. 2023 Aug 18;25:e41805. doi: 10.2196/41805 (PMC10474518; doi:10.2196/41805)
Supplement: Multimedia Appendix 1 [file jmir_v25i1e41805_app1.docx]

**Appendix 1. Scenarios**

**Scenario 1:**

Dear participants,

Please read the provided definition and case scenario first and then answer the following questions accordingly.

**Scenario:**

This study is designed to understand your opinions about using new technology for sharing your digital information. This technology allows you to be part of a distributed network for accessing data and information sharing. These distributed networks used for sharing information are called Blockchain-based solutions. In this model, each member in the network stores an identical copy and contributes to the collective process of validating and certifying digital transactions of data in the network. Each member of the network can oversee the process of data sharing across the network. Blockchain-based technology can be used for sharing healthcare information, financial information, or any other digital records. In this network, users can manage data sharing by controlling how they can access and view their personal digital information.

Imagine a Blockchain-based technology (such as the online network defined above) is available for you to share your sensitive health information with researchers and scientists in a secured network for clinical research purposes. The company that provides this technology is called Company X-Block:

This company defines its offerings as follows:

General features:

- A private network to mediate the searching, storing, buying, and selling of health data

- Individuals may choose to allow scientists to search for and buy their data

- Putting the individual in charge of his/her data, and individuals can consent to how their data is used in research

- Anyone is free to safely store their data on the companies' network chain

- There is no personal identifiers to data, and it will be de-identified for sharing purposes

- Data donors (individuals participating in this network) can remote their health information from the network if they are no longer interested in sharing data.

The main difference between this company compared to similar network-based solutions offered by other companies is:

Special feature:

They incentivize individuals to share data by offering **cryptocurrency**(digital currency).

Some notes:

- A cryptocurrency is a type of [digital](https://simple.wikipedia.org/wiki/Currency) money that is controlled by a network of computers and removes the need for intermediaries to validate transactions. The list of all transactions is shared publicly through a public ledger. People can buy products/services and pay online using their cryptocurrencies. However, individuals’ real identities do not appear in the public ledger.

- Each individual who gives permission to researchers will receive shares in the form of cryptocurrency

-The offered cryptocurrency can be exchangeable with other cryptocurrencies (such as Bitcoin and Ethereum)

Now, please answer the following questions based on the given scenario. Thanks. 

**Scenario 2:**

Dear participants,

Please read the provided definition and case scenario first and then answer the following questions accordingly.

**Scenario:**

This study is designed to understand your opinions about using new technology for sharing your digital information. This technology allows you to be part of a distributed network for accessing data and information sharing. These distributed networks used for sharing information are called Blockchain-based solutions. In this model, each member in the network stores an identical copy and contributes to the collective process of validating and certifying digital transactions of data in the network. Each member of the network can oversee the process of data sharing across the network. Blockchain-based technology can be used for sharing healthcare information, financial information, or any other digital records. In this network, users can manage data sharing by controlling how they can access and view their personal digital information.

Imagine a Blockchain-based technology (such as the online network defined above) is available for you to share your sensitive health information with researchers and scientists in a secured network for clinical research purposes. The company that provides this technology is called Company X-Block:

This company defines its offerings as follows:

General features:

- A private network to mediate the searching, storing, buying, and selling of health data

- Individuals may choose to allow scientists to search for and buy their data

- Putting the individual in charge of his/her data, and individuals can consent to how their data is used in research

- Anyone is free to safely store their data on the companies' network chain

- There are no personal identifiers to data, and it will be de-identified for sharing purposes

- Data donors (individuals participating in this network) can remote their health information from the network if they are no longer interested in sharing data.

The main difference between this company compared to similar network-based solutions offered by other companies is:

Special feature:

They incentivize individuals to share data by offering **money**(any currency)

- Each individual who gives permission to researchers to use his/her health information for clinical research will receive shares in the form of money

Now, please answer the following questions based on the given scenario. Thanks.

**Scenario 3:**

Dear participants,

Please read the provided definition and case scenario first and then answer the following questions accordingly.

**Scenario:**

This study is designed to understand your opinions about using new technology for sharing your digital information. This technology allows you to be part of a distributed network for accessing data and information sharing. These distributed networks used for sharing information are called Blockchain-based solutions. In this model, each member in the network stores an identical copy and contributes to the collective process of validating and certifying digital transactions of data in the network. Each member of the network can oversee the process of data sharing across the network. Blockchain-based technology can be used for sharing healthcare information, financial information, or any other digital records. In this network, users can manage data sharing by controlling how they can access and view their personal digital information.

Imagine a Blockchain-based technology (such as the online network defined above) is available for you to share your sensitive health information with researchers and scientists in a secured network for clinical research purposes. The company that provides this technology is called Company X-Block:

This company defines its offerings as follows:

General features:

- A private network to mediate the searching, storing, buying, and selling of health data

- Individuals may choose to allow scientists to search for and buy their data

- Putting the individual in charge of his/her data, and individuals can consent to how their data is used in research

- Anyone is free to safely store their data on the companies' network chain

- There is no personal identifiers to data, and it will be de-identified for sharing purposes

- Data donors (individuals participating in this network) can remote their health information from the network if they are no longer interested in sharing data.

The main difference between this company compared to similar network-based solutions offered by other companies is:

They incentivize individuals to share data by offering **academic credits and recognition points**

- Each individual who gives permission to researchers will receive shares in the form of **Authorship credits**

-The Authorship credits will be integrated into the Blockchain-based platforms and shared with others. 

- The holders of the Authorship credits will receive recognition for sharing their health data that could be used for clinical research purposes to:

1. Improve public healthcare quality
2. Enhance the quality of healthcare findings 
3. Reducing health risk factors 
4. Identify the best healthcare practices
5. Discover new treatment and care planning 

Now, please answer the following questions based on the given scenario. Thanks.
