# Supplementary material for: Role of Incentives in the Use of Blockchain-Based Platforms for Sharing Sensitive Health Data: Experimental Study
Source: J Med Internet Res. 2023 Aug 18;25:e41805. doi: 10.2196/41805 (PMC10474518; doi:10.2196/41805)
Supplement: Multimedia Appendix 2 [file jmir_v25i1e41805_app2.docx]

**Appendix 2. Measurement items (shared across the three scenarios)**

Considering the case scenario of using Blockchain-based solutions with incentives, please answer the following questions:

**Perceived efficiency of data sharing:**

- I think using this system can speed up the data-sharing process
- I think it is convenient for me to use this system for data sharing
- I believe this system can effectively lower the hassles of sharing health-related data
- I think this system can efficiently connect data holders and data buyers
- I think this system can automate data-sharing procedures
- Overall, I think using this system will increase the efficiency of data sharing

**Perceived trust in the competency of the network:**

- I trust in the capability of this system to share data with researchers
- I trust in the ability of this system's technology in the data-sharing process
- I trust that this system has a standard of competency to carry out data sharing
- I trust that this system is a real expert system to use for data sharing
- I trust that this system is a reliable technology to use for information sharing with researchers

**Perceived transparency:**

- I believe that this system makes the purpose of data sharing clear
- I believe that this system makes it transparent what information is shared and used
- I believe that this system makes the procedure of data sharing transparent
- I think that this system makes the consent mechanism clear
- I believe that this system increases the transparency of the data access management

**Perceived control:**

- I think using this system; I can oversee data access requests and approvals
- I think using this system; I am part of the access control
- I think using this system; I can control the authorization process
- I believe using this system; I am involved in a participatory access control
- I think using this system; I can maintain ongoing control of my own health data

**Perceived incentives:**

- I think the incentivizing mechanism used in this system is very helpful
- I believe offering the incentives (which are explained in the above scenario) will encourage me to store and share my health-related data with this system
- I think offering the incentives (which are explained in the above scenario) is useful to reward data-sharing efforts
- I think it is important to provide the incentives (which are explained in the above scenario) to share data by offering cryptocurrency
- Overall, I think by offering the incentives (which are explained in the above scenario), I will be incentivized to share my health data for research purposes

**Perceived anonymity:**

- I believe this system will enhance the anonymity of data sharing
- I think it is important that this system provides anonymized health data
- I believe this system can help me release de-identified sensitive data to research organizations that ask for it
- I think this system can anonymously offer health data to pharmaceutical and research companies
- Overall, I think it is important that this system removes personal identifiers from sensitive health data for sharing purposes

**Perceived privacy concern:**

- I think using this system, researchers or public health authorities may collect too much personal information from me
- I think using this system, researchers or public health authorities may use my information for other purposes without my knowledge and authorization
- I think using this system; my health information will be shared with other researchers or public health authorities without my explicit consent
- I think using this system; unauthorized people will have access to my health information
- I feel concerned about the privacy of my health information using this system
- I think using this system; my health information may be sold to others without my permission

**Perceived data ownership**

- I believe that this system can increase data ownership
- I think this system entails data stewardship
- I believe using this system; I get access to my raw health data
- I think this system recognizes the ownership rights of my raw health data
- I think using this system; I could retain ownership of my personal health-related data

**Willingness to Use**

- I agree to use this system for data-sharing purposes
- I think using this system for sharing health data is something I would consider
- I would like to use this system to manage data sharing with researchers
- In the future, I am willing to use this system for data sharing
- I am very likely to use this system for sharing my raw health data with research companies

**Familiarity with Blockchain system (prior experience):**

- Based on previous experience, I am familiar with how this system performs
- In general, I am familiar with the process of data sharing through this system
- In the past, I have used this system to share my health data
- I am familiar with how this system helps research companies access raw health data
- Overall, I am familiar with this system’s mechanism for sharing purposes

**Demographics:**

Gender

**Male**

**Female**

Age

**Under 20**

**20-29**

**30-39**

**40-49**

**50-59**

**60 or older**

Annual household income

**Less than $25,000**

**$25,000-$49,999**

**$50,000-$74,999**

**$75,000-$99,999**

**$100,000 or $150,000**

**More than $150,000**

What category best describes your level of education?

**Less than high school**

**High school graduate**

**Some college**

**2-year degree**

**Bachelor's degree**

**Master's degree**

**Doctorate**

Employment status?

**Employed full time**

**Employed part-time**

**Unemployed**

**Retired**

**Student**

Race/Ethnicity

**White**

**African American**

**Asian**

**Hispanic**

**Mixed**

**Other**

In your opinion, what are the barriers to using this Blockchain technology to share sensitive health data? (You can select more than one option)

- Lack of knowledge and familiarity with this technology
- Lack of trust in the technology
- Privacy and security concerns
- Lack of public acceptance
- Lack of regulations to support this technology
- This technology is still not available in hospitals
- The use of this technology may increase my healthcare costs
- I think this technology is complicated
- My physicians do not use this technology in their practices
- I do not believe in cryptocurrency

Besides the proposed incentive in this scenario, please explain what else can motivate you to use this technology for sharing your health data in the future.
