# Supplementary material for: Role of Incentives in the Use of Blockchain-Based Platforms for Sharing Sensitive Health Data: Experimental Study
Source: J Med Internet Res. 2023 Aug 18;25:e41805. doi: 10.2196/41805 (PMC10474518; doi:10.2196/41805)
Supplement: Multimedia Appendix 3 [file jmir_v25i1e41805_app3.docx]

**Appendix 3.** **Results of Scheffe post hoc analysis**

| Dependent Variable | Scenarios(I) | Scenario(J) | Mean Difference (I-J) | Std. Error | p_value | 95% Confidence Interval | |
| --- | --- | --- | --- | --- | --- | --- | --- |
|  |  |  |  |  |  | Lower Bound | Upper Bound |
| Willingness | Cryptocurrency/Less familiar | Money/Less familiar | -1.219 | 0.805 | 0.807 | -3.91 | 1.47 |
|  |  | Recognition/Less familiar | 1.358 | 0.690 | 0.569 | -0.95 | 3.66 |
|  |  | Cryptocurrency/Highly familiar | -4.684^*^ | 0.596 | 0.000 | -6.68 | -2.69 |
|  |  | Money/Highly familiar | -4.544^*^ | 0.570 | 0.000 | -6.45 | -2.64 |
|  |  | Recognition/Highly familiar | -4.957^*^ | 0.577 | 0.000 | -6.88 | -3.03 |
|  | Money/Less familiar | Cryptocurrency/Less familiar | 1.219 | 0.805 | 0.807 | -1.47 | 3.91 |
|  |  | Recognition/Less familiar | 2.577 | 0.832 | 0.090 | -0.20 | 5.36 |
|  |  | Cryptocurrency/Highly familiar | -3.465^*^ | 0.756 | 0.001 | -5.99 | -0.94 |
|  |  | Money/Highly familiar | -3.325^*^ | 0.735 | 0.001 | -5.78 | -0.87 |
|  |  | Recognition/Highly familiar | -3.738^*^ | 0.741 | 0.000 | -6.21 | -1.26 |
|  | Recognition/Less familiar | Cryptocurrency/Less familiar | -1.358 | 0.690 | 0.569 | -3.66 | 0.95 |
|  |  | Money/Less familiar | -2.577 | 0.832 | 0.090 | -5.36 | 0.20 |
|  |  | Cryptocurrency/Highly familiar | -6.042^*^ | 0.633 | 0.000 | -8.16 | -3.93 |
|  |  | Money/Highly familiar | -5.902^*^ | 0.608 | 0.000 | -7.93 | -3.87 |
|  |  | Recognition/Highly familiar | -6.315^*^ | 0.615 | 0.000 | -8.37 | -4.26 |
|  | Cryptocurrency/Highly familiar | Cryptocurrency/Less familiar | 4.684^*^ | 0.596 | 0.000 | 2.69 | 6.68 |
|  |  | Money/Less familiar | 3.465^*^ | 0.756 | 0.001 | 0.94 | 5.99 |
|  |  | Recognition/Less familiar | 6.042^*^ | 0.633 | 0.000 | 3.93 | 8.16 |
|  |  | Money/Highly familiar | 0.140 | 0.498 | 1.000 | -1.52 | 1.80 |
|  |  | Recognition/Highly familiar | -0.273 | 0.507 | 0.998 | -1.97 | 1.42 |
|  | Money/Highly familiar | Cryptocurrency/Less familiar | 4.544^*^ | 0.570 | 0.000 | 2.64 | 6.45 |
|  |  | Money/Less familiar | 3.325^*^ | 0.735 | 0.001 | 0.87 | 5.78 |
|  |  | Recognition/Less familiar | 5.902^*^ | 0.608 | 0.000 | 3.87 | 7.93 |
|  |  | Cryptocurrency/Highly familiar | -0.140 | 0.498 | 1.000 | -1.80 | 1.52 |
|  |  | Recognition/Highly familiar | -0.413 | 0.475 | 0.980 | -2.00 | 1.17 |
|  | Recognition/Highly familiar | Cryptocurrency/Less familiar | 4.957^*^ | 0.577 | 0.000 | 3.03 | 6.88 |
|  |  | Money/Less familiar | 3.738^*^ | 0.741 | 0.000 | 1.26 | 6.21 |
|  |  | Recognition/Less familiar | 6.315^*^ | 0.615 | 0.000 | 4.26 | 8.37 |
|  |  | Cryptocurrency/Highly familiar | 0.273 | 0.507 | 0.998 | -1.42 | 1.97 |
|  |  | Money/Highly familiar | 0.413 | 0.475 | 0.980 | -1.17 | 2.00 |
| Data Ownership | Cryptocurrency/Less familiar | Money/Less familiar | -0.422 | 0.690 | 0.996 | -2.73 | 1.88 |
|  |  | Recognition/Less familiar | 0.514 | 0.592 | 0.980 | -1.46 | 2.49 |
|  |  | Cryptocurrency/Highly familiar | -4.075^*^ | 0.511 | 0.000 | -5.78 | -2.37 |
|  |  | Money/Highly familiar | -3.809^*^ | 0.488 | 0.000 | -5.44 | -2.18 |
|  |  | Recognition/Highly familiar | -4.416^*^ | 0.495 | 0.000 | -6.07 | -2.76 |
|  | Money/Less familiar | Cryptocurrency/Less familiar | 0.422 | 0.690 | 0.996 | -1.88 | 2.73 |
|  |  | Recognition/Less familiar | 0.936 | 0.713 | 0.886 | -1.45 | 3.32 |
|  |  | Cryptocurrency/Highly familiar | -3.653^*^ | 0.648 | 0.000 | -5.82 | -1.49 |
|  |  | Money/Highly familiar | -3.387^*^ | 0.630 | 0.000 | -5.49 | -1.28 |
|  |  | Recognition/Highly familiar | -3.994^*^ | 0.635 | 0.000 | -6.12 | -1.87 |
|  | Recognition/Less familiar | Cryptocurrency/Less familiar | -0.514 | 0.592 | 0.980 | -2.49 | 1.46 |
|  |  | Money/Less familiar | -0.936 | 0.713 | 0.886 | -3.32 | 1.45 |
|  |  | Cryptocurrency/Highly familiar | -4.589^*^ | 0.542 | 0.000 | -6.40 | -2.78 |
|  |  | Money/Highly familiar | -4.323^*^ | 0.521 | 0.000 | -6.06 | -2.58 |
|  |  | Recognition/Highly familiar | -4.930^*^ | 0.527 | 0.000 | -6.69 | -3.17 |
|  | Cryptocurrency/Highly familiar | Cryptocurrency/Less familiar | 4.075^*^ | 0.511 | 0.000 | 2.37 | 5.78 |
|  |  | Money/Less familiar | 3.653^*^ | 0.648 | 0.000 | 1.49 | 5.82 |
|  |  | Recognition/Less familiar | 4.589^*^ | 0.542 | 0.000 | 2.78 | 6.40 |
|  |  | Money/Highly familiar | 0.266 | 0.427 | 0.996 | -1.16 | 1.69 |
|  |  | Recognition/Highly familiar | -0.341 | 0.434 | 0.987 | -1.79 | 1.11 |
|  | Money/Highly familiar | Cryptocurrency/Less familiar | 3.809^*^ | 0.488 | 0.000 | 2.18 | 5.44 |
|  |  | Money/Less familiar | 3.387^*^ | 0.630 | 0.000 | 1.28 | 5.49 |
|  |  | Recognition/Less familiar | 4.323^*^ | 0.521 | 0.000 | 2.58 | 6.06 |
|  |  | Cryptocurrency/Highly familiar | -0.266 | 0.427 | 0.996 | -1.69 | 1.16 |
|  |  | Recognition/Highly familiar | -0.607 | 0.407 | 0.818 | -1.97 | 0.75 |
|  | Recognition/Highly familiar | Cryptocurrency/Less familiar | 4.416^*^ | 0.495 | 0.000 | 2.76 | 6.07 |
|  |  | Money/Less familiar | 3.994^*^ | 0.635 | 0.000 | 1.87 | 6.12 |
|  |  | Recognition/Less familiar | 4.930^*^ | 0.527 | 0.000 | 3.17 | 6.69 |
|  |  | Cryptocurrency/Highly familiar | 0.341 | 0.434 | 0.987 | -1.11 | 1.79 |
|  |  | Money/Highly familiar | 0.607 | 0.407 | 0.818 | -0.75 | 1.97 |
| Privacy | Cryptocurrency/Less familiar | Money/Less familiar | 1.219 | 1.089 | 0.939 | -2.42 | 4.86 |
|  |  | Recognition/Less familiar | 0.657 | 0.934 | 0.992 | -2.46 | 3.78 |
|  |  | Cryptocurrency/Highly familiar | -0.905 | 0.807 | 0.939 | -3.60 | 1.79 |
|  |  | Money/Highly familiar | -3.161^*^ | 0.771 | 0.005 | -5.74 | -0.59 |
|  |  | Recognition/Highly familiar | -3.523^*^ | 0.781 | 0.001 | -6.13 | -0.91 |
|  | Money/Less familiar | Cryptocurrency/Less familiar | -1.219 | 1.089 | 0.939 | -4.86 | 2.42 |
|  |  | Recognition/Less familiar | -0.561 | 1.126 | 0.998 | -4.32 | 3.20 |
|  |  | Cryptocurrency/Highly familiar | -2.124 | 1.023 | 0.506 | -5.54 | 1.29 |
|  |  | Money/Highly familiar | -4.380^*^ | 0.995 | 0.002 | -7.70 | -1.06 |
|  |  | Recognition/Highly familiar | -4.742^*^ | 1.002 | 0.001 | -8.09 | -1.39 |
|  | Recognition/Less familiar | Cryptocurrency/Less familiar | -0.657 | 0.934 | 0.992 | -3.78 | 2.46 |
|  |  | Money/Less familiar | 0.561 | 1.126 | 0.998 | -3.20 | 4.32 |
|  |  | Cryptocurrency/Highly familiar | -1.562 | 0.856 | 0.649 | -4.42 | 1.30 |
|  |  | Money/Highly familiar | -3.819^*^ | 0.822 | 0.001 | -6.57 | -1.07 |
|  |  | Recognition/Highly familiar | -4.180^*^ | 0.832 | 0.000 | -6.96 | -1.40 |
|  | Cryptocurrency/Highly familiar | Cryptocurrency/Less familiar | 0.905 | 0.807 | 0.939 | -1.79 | 3.60 |
|  |  | Money/Less familiar | 2.124 | 1.023 | 0.506 | -1.29 | 5.54 |
|  |  | Recognition/Less familiar | 1.562 | 0.856 | 0.649 | -1.30 | 4.42 |
|  |  | Money/Highly familiar | -2.256^*^ | 0.674 | 0.049 | -4.51 | 0.00 |
|  |  | Recognition/Highly familiar | -2.618^*^ | 0.685 | 0.013 | -4.91 | -0.33 |
|  | Money/Highly familiar | Cryptocurrency/Less familiar | 3.161^*^ | 0.771 | 0.005 | 0.59 | 5.74 |
|  |  | Money/Less familiar | 4.380^*^ | 0.995 | 0.002 | 1.06 | 7.70 |
|  |  | Recognition/Less familiar | 3.819^*^ | 0.822 | 0.001 | 1.07 | 6.57 |
|  |  | Cryptocurrency/Highly familiar | 2.256^*^ | 0.674 | 0.049 | 0.00 | 4.51 |
|  |  | Recognition/Highly familiar | -0.362 | 0.643 | 0.997 | -2.51 | 1.79 |
|  | Recognition/Highly familiar | Cryptocurrency/Less familiar | 3.523^*^ | 0.781 | 0.001 | 0.91 | 6.13 |
|  |  | Money/Less familiar | 4.742^*^ | 1.002 | 0.001 | 1.39 | 8.09 |
|  |  | Recognition/Less familiar | 4.180^*^ | 0.832 | 0.000 | 1.40 | 6.96 |
|  |  | Cryptocurrency/Highly familiar | 2.618^*^ | 0.685 | 0.013 | 0.33 | 4.91 |
|  |  | Money/Highly familiar | 0.362 | 0.643 | 0.997 | -1.79 | 2.51 |
| Anonymity | Cryptocurrency/Less familiar | Money/Less familiar | -0.438 | 0.752 | 0.997 | -2.95 | 2.08 |
|  |  | Recognition/Less familiar | 0.780 | 0.645 | 0.917 | -1.38 | 2.94 |
|  |  | Cryptocurrency/Highly familiar | -3.344^*^ | 0.557 | 0.000 | -5.21 | -1.48 |
|  |  | Money/Highly familiar | -2.984^*^ | 0.533 | 0.000 | -4.76 | -1.20 |
|  |  | Recognition/Highly familiar | -3.124^*^ | 0.540 | 0.000 | -4.93 | -1.32 |
|  | Money/Less familiar | Cryptocurrency/Less familiar | 0.438 | 0.752 | 0.997 | -2.08 | 2.95 |
|  |  | Recognition/Less familiar | 1.218 | 0.778 | 0.784 | -1.38 | 3.82 |
|  |  | Cryptocurrency/Highly familiar | -2.907^*^ | 0.707 | 0.005 | -5.27 | -0.55 |
|  |  | Money/Highly familiar | -2.547^*^ | 0.687 | 0.019 | -4.84 | -0.25 |
|  |  | Recognition/Highly familiar | -2.687^*^ | 0.693 | 0.011 | -5.00 | -0.37 |
|  | Recognition/Less familiar | Cryptocurrency/Less familiar | -0.780 | 0.645 | 0.917 | -2.94 | 1.38 |
|  |  | Money/Less familiar | -1.218 | 0.778 | 0.784 | -3.82 | 1.38 |
|  |  | Cryptocurrency/Highly familiar | -4.124^*^ | 0.592 | 0.000 | -6.10 | -2.15 |
|  |  | Money/Highly familiar | -3.764^*^ | 0.568 | 0.000 | -5.66 | -1.87 |
|  |  | Recognition/Highly familiar | -3.905^*^ | 0.575 | 0.000 | -5.82 | -1.98 |
|  | Cryptocurrency/Highly familiar | Cryptocurrency/Less familiar | 3.344^*^ | 0.557 | 0.000 | 1.48 | 5.21 |
|  |  | Money/Less familiar | 2.907^*^ | 0.707 | 0.005 | 0.55 | 5.27 |
|  |  | Recognition/Less familiar | 4.124^*^ | 0.592 | 0.000 | 2.15 | 6.10 |
|  |  | Money/Highly familiar | 0.360 | 0.466 | 0.988 | -1.20 | 1.92 |
|  |  | Recognition/Highly familiar | 0.220 | 0.474 | 0.999 | -1.36 | 1.80 |
|  | Money/Highly familiar | Cryptocurrency/Less familiar | 2.984^*^ | 0.533 | 0.000 | 1.20 | 4.76 |
|  |  | Money/Less familiar | 2.547^*^ | 0.687 | 0.019 | 0.25 | 4.84 |
|  |  | Recognition/Less familiar | 3.764^*^ | 0.568 | 0.000 | 1.87 | 5.66 |
|  |  | Cryptocurrency/Highly familiar | -0.360 | 0.466 | 0.988 | -1.92 | 1.20 |
|  |  | Recognition/Highly familiar | -0.140 | 0.444 | 1.000 | -1.63 | 1.34 |
|  | Recognition/Highly familiar | Cryptocurrency/Less familiar | 3.124^*^ | 0.540 | 0.000 | 1.32 | 4.93 |
|  |  | Money/Less familiar | 2.687^*^ | 0.693 | 0.011 | 0.37 | 5.00 |
|  |  | Recognition/Less familiar | 3.905^*^ | 0.575 | 0.000 | 1.98 | 5.82 |
|  |  | Cryptocurrency/Highly familiar | -0.220 | 0.474 | 0.999 | -1.80 | 1.36 |
|  |  | Money/Highly familiar | 0.140 | 0.444 | 1.000 | -1.34 | 1.63 |
| Incentives | Cryptocurrency/Less familiar | Money/Less familiar | -0.984 | 0.824 | 0.921 | -3.74 | 1.77 |
|  |  | Recognition/Less familiar | 0.999 | 0.707 | 0.849 | -1.36 | 3.36 |
|  |  | Cryptocurrency/Highly familiar | -4.491^*^ | 0.611 | 0.000 | -6.53 | -2.45 |
|  |  | Money/Highly familiar | -4.115^*^ | 0.584 | 0.000 | -6.07 | -2.17 |
|  |  | Recognition/Highly familiar | -4.317^*^ | 0.591 | 0.000 | -6.29 | -2.34 |
|  | Money/Less familiar | Cryptocurrency/Less familiar | 0.984 | 0.824 | 0.921 | -1.77 | 3.74 |
|  |  | Recognition/Less familiar | 1.983 | 0.852 | 0.369 | -0.86 | 4.83 |
|  |  | Cryptocurrency/Highly familiar | -3.506^*^ | 0.774 | 0.001 | -6.09 | -0.92 |
|  |  | Money/Highly familiar | -3.131^*^ | 0.753 | 0.004 | -5.65 | -0.62 |
|  |  | Recognition/Highly familiar | -3.333^*^ | 0.759 | 0.002 | -5.87 | -0.80 |
|  | Recognition/Less familiar | Cryptocurrency/Less familiar | -0.999 | 0.707 | 0.849 | -3.36 | 1.36 |
|  |  | Money/Less familiar | -1.983 | 0.852 | 0.369 | -4.83 | 0.86 |
|  |  | Cryptocurrency/Highly familiar | -5.490^*^ | 0.648 | 0.000 | -7.65 | -3.32 |
|  |  | Money/Highly familiar | -5.114^*^ | 0.623 | 0.000 | -7.19 | -3.03 |
|  |  | Recognition/Highly familiar | -5.316^*^ | 0.629 | 0.000 | -7.42 | -3.21 |
|  | Cryptocurrency/Highly familiar | Cryptocurrency/Less familiar | 4.491^*^ | 0.611 | 0.000 | 2.45 | 6.53 |
|  |  | Money/Less familiar | 3.506^*^ | 0.774 | 0.001 | 0.92 | 6.09 |
|  |  | Recognition/Less familiar | 5.490^*^ | 0.648 | 0.000 | 3.32 | 7.65 |
|  |  | Money/Highly familiar | 0.375 | 0.510 | 0.990 | -1.33 | 2.08 |
|  |  | Recognition/Highly familiar | 0.174 | 0.519 | 1.000 | -1.56 | 1.91 |
|  | Money/Highly familiar | Cryptocurrency/Less familiar | 4.115^*^ | 0.584 | 0.000 | 2.17 | 6.07 |
|  |  | Money/Less familiar | 3.131^*^ | 0.753 | 0.004 | 0.62 | 5.65 |
|  |  | Recognition/Less familiar | 5.114^*^ | 0.623 | 0.000 | 3.03 | 7.19 |
|  |  | Cryptocurrency/Highly familiar | -0.375 | 0.510 | 0.990 | -2.08 | 1.33 |
|  |  | Recognition/Highly familiar | -0.202 | 0.487 | 0.999 | -1.83 | 1.42 |
|  | Recognition/Highly familiar | Cryptocurrency/Less familiar | 4.317^*^ | 0.591 | 0.000 | 2.34 | 6.29 |
|  |  | Money/Less familiar | 3.333^*^ | 0.759 | 0.002 | 0.80 | 5.87 |
|  |  | Recognition/Less familiar | 5.316^*^ | 0.629 | 0.000 | 3.21 | 7.42 |
|  |  | Cryptocurrency/Highly familiar | -0.174 | 0.519 | 1.000 | -1.91 | 1.56 |
|  |  | Money/Highly familiar | 0.202 | 0.487 | 0.999 | -1.42 | 1.83 |
| Control | Cryptocurrency/Less familiar | Money/Less familiar | -0.375 | 0.778 | 0.999 | -2.98 | 2.23 |
|  |  | Recognition/Less familiar | -0.164 | 0.668 | 1.000 | -2.39 | 2.07 |
|  |  | Cryptocurrency/Highly familiar | -3.604^*^ | 0.577 | 0.000 | -5.53 | -1.68 |
|  |  | Money/Highly familiar | -3.725^*^ | 0.551 | 0.000 | -5.57 | -1.88 |
|  |  | Recognition/Highly familiar | -4.005^*^ | 0.558 | 0.000 | -5.87 | -2.14 |
|  | Money/Less familiar | Cryptocurrency/Less familiar | 0.375 | 0.778 | 0.999 | -2.23 | 2.98 |
|  |  | Recognition/Less familiar | 0.211 | 0.805 | 1.000 | -2.48 | 2.90 |
|  |  | Cryptocurrency/Highly familiar | -3.229^*^ | 0.731 | 0.002 | -5.67 | -0.79 |
|  |  | Money/Highly familiar | -3.350^*^ | 0.711 | 0.001 | -5.73 | -0.97 |
|  |  | Recognition/Highly familiar | -3.630^*^ | 0.716 | 0.000 | -6.02 | -1.24 |
|  | Recognition/Less familiar | Cryptocurrency/Less familiar | 0.164 | 0.668 | 1.000 | -2.07 | 2.39 |
|  |  | Money/Less familiar | -0.211 | 0.805 | 1.000 | -2.90 | 2.48 |
|  |  | Cryptocurrency/Highly familiar | -3.440^*^ | 0.612 | 0.000 | -5.48 | -1.40 |
|  |  | Money/Highly familiar | -3.561^*^ | 0.588 | 0.000 | -5.53 | -1.60 |
|  |  | Recognition/Highly familiar | -3.841^*^ | 0.594 | 0.000 | -5.83 | -1.86 |
|  | Cryptocurrency/Highly familiar | Cryptocurrency/Less familiar | 3.604^*^ | 0.577 | 0.000 | 1.68 | 5.53 |
|  |  | Money/Less familiar | 3.229^*^ | 0.731 | 0.002 | 0.79 | 5.67 |
|  |  | Recognition/Less familiar | 3.440^*^ | 0.612 | 0.000 | 1.40 | 5.48 |
|  |  | Money/Highly familiar | -0.121 | 0.482 | 1.000 | -1.73 | 1.49 |
|  |  | Recognition/Highly familiar | -0.401 | 0.490 | 0.984 | -2.04 | 1.24 |
|  | Money/Highly familiar | Cryptocurrency/Less familiar | 3.725^*^ | 0.551 | 0.000 | 1.88 | 5.57 |
|  |  | Money/Less familiar | 3.350^*^ | 0.711 | 0.001 | 0.97 | 5.73 |
|  |  | Recognition/Less familiar | 3.561^*^ | 0.588 | 0.000 | 1.60 | 5.53 |
|  |  | Cryptocurrency/Highly familiar | 0.121 | 0.482 | 1.000 | -1.49 | 1.73 |
|  |  | Recognition/Highly familiar | -0.280 | 0.460 | 0.996 | -1.82 | 1.26 |
|  | Recognition/Highly familiar | Cryptocurrency/Less familiar | 4.005^*^ | 0.558 | 0.000 | 2.14 | 5.87 |
|  |  | Money/Less familiar | 3.630^*^ | 0.716 | 0.000 | 1.24 | 6.02 |
|  |  | Recognition/Less familiar | 3.841^*^ | 0.594 | 0.000 | 1.86 | 5.83 |
|  |  | Cryptocurrency/Highly familiar | 0.401 | 0.490 | 0.984 | -1.24 | 2.04 |
|  |  | Money/Highly familiar | 0.280 | 0.460 | 0.996 | -1.26 | 1.82 |
| Transparency | Cryptocurrency/Less familiar | Money/Less familiar | -1.813 | 0.809 | 0.414 | -4.51 | 0.89 |
|  |  | Recognition/Less familiar | 0.163 | 0.694 | 1.000 | -2.15 | 2.48 |
|  |  | Cryptocurrency/Highly familiar | -3.633^*^ | 0.599 | 0.000 | -5.63 | -1.63 |
|  |  | Money/Highly familiar | -3.505^*^ | 0.573 | 0.000 | -5.42 | -1.59 |
|  |  | Recognition/Highly familiar | -3.536^*^ | 0.580 | 0.000 | -5.47 | -1.60 |
|  | Money/Less familiar | Cryptocurrency/Less familiar | 1.813 | 0.809 | 0.414 | -0.89 | 4.51 |
|  |  | Recognition/Less familiar | 1.975 | 0.836 | 0.351 | -0.82 | 4.77 |
|  |  | Cryptocurrency/Highly familiar | -1.820 | 0.759 | 0.334 | -4.36 | 0.72 |
|  |  | Money/Highly familiar | -1.692 | 0.739 | 0.388 | -4.16 | 0.78 |
|  |  | Recognition/Highly familiar | -1.724 | 0.744 | 0.375 | -4.21 | 0.76 |
|  | Recognition/Less familiar | Cryptocurrency/Less familiar | -0.163 | 0.694 | 1.000 | -2.48 | 2.15 |
|  |  | Money/Less familiar | -1.975 | 0.836 | 0.351 | -4.77 | 0.82 |
|  |  | Cryptocurrency/Highly familiar | -3.795^*^ | 0.636 | 0.000 | -5.92 | -1.67 |
|  |  | Money/Highly familiar | -3.668^*^ | 0.611 | 0.000 | -5.71 | -1.63 |
|  |  | Recognition/Highly familiar | -3.699^*^ | 0.618 | 0.000 | -5.76 | -1.64 |
|  | Cryptocurrency/Highly familiar | Cryptocurrency/Less familiar | 3.633^*^ | 0.599 | 0.000 | 1.63 | 5.63 |
|  |  | Money/Less familiar | 1.820 | 0.759 | 0.334 | -0.72 | 4.36 |
|  |  | Recognition/Less familiar | 3.795^*^ | 0.636 | 0.000 | 1.67 | 5.92 |
|  |  | Money/Highly familiar | 0.128 | 0.501 | 1.000 | -1.55 | 1.80 |
|  |  | Recognition/Highly familiar | 0.097 | 0.509 | 1.000 | -1.60 | 1.80 |
|  | Money/Highly familiar | Cryptocurrency/Less familiar | 3.505^*^ | 0.573 | 0.000 | 1.59 | 5.42 |
|  |  | Money/Less familiar | 1.692 | 0.739 | 0.388 | -0.78 | 4.16 |
|  |  | Recognition/Less familiar | 3.668^*^ | 0.611 | 0.000 | 1.63 | 5.71 |
|  |  | Cryptocurrency/Highly familiar | -0.128 | 0.501 | 1.000 | -1.80 | 1.55 |
|  |  | Recognition/Highly familiar | -0.031 | 0.478 | 1.000 | -1.63 | 1.56 |
|  | Recognition/Highly familiar | Cryptocurrency/Less familiar | 3.536^*^ | 0.580 | 0.000 | 1.60 | 5.47 |
|  |  | Money/Less familiar | 1.724 | 0.744 | 0.375 | -0.76 | 4.21 |
|  |  | Recognition/Less familiar | 3.699^*^ | 0.618 | 0.000 | 1.64 | 5.76 |
|  |  | Cryptocurrency/Highly familiar | -0.097 | 0.509 | 1.000 | -1.80 | 1.60 |
|  |  | Money/Highly familiar | 0.031 | 0.478 | 1.000 | -1.56 | 1.63 |
| Trust | Cryptocurrency/Less familiar | Money/Less familiar | -0.953 | 0.804 | 0.923 | -3.64 | 1.73 |
|  |  | Recognition/Less familiar | 0.978 | 0.690 | 0.847 | -1.33 | 3.28 |
|  |  | Cryptocurrency/Highly familiar | -4.098^*^ | 0.596 | 0.000 | -6.09 | -2.11 |
|  |  | Money/Highly familiar | -3.645^*^ | 0.569 | 0.000 | -5.55 | -1.74 |
|  |  | Recognition/Highly familiar | -3.782^*^ | 0.577 | 0.000 | -5.71 | -1.86 |
|  | Money/Less familiar | Cryptocurrency/Less familiar | 0.953 | 0.804 | 0.923 | -1.73 | 3.64 |
|  |  | Recognition/Less familiar | 1.932 | 0.831 | 0.371 | -0.85 | 4.71 |
|  |  | Cryptocurrency/Highly familiar | -3.145^*^ | 0.755 | 0.004 | -5.67 | -0.62 |
|  |  | Money/Highly familiar | -2.692^*^ | 0.735 | 0.021 | -5.15 | -0.24 |
|  |  | Recognition/Highly familiar | -2.828^*^ | 0.740 | 0.013 | -5.30 | -0.36 |
|  | Recognition/Less familiar | Cryptocurrency/Less familiar | -0.978 | 0.690 | 0.847 | -3.28 | 1.33 |
|  |  | Money/Less familiar | -1.932 | 0.831 | 0.371 | -4.71 | 0.85 |
|  |  | Cryptocurrency/Highly familiar | -5.077^*^ | 0.632 | 0.000 | -7.19 | -2.96 |
|  |  | Money/Highly familiar | -4.624^*^ | 0.607 | 0.000 | -6.65 | -2.59 |
|  |  | Recognition/Highly familiar | -4.760^*^ | 0.614 | 0.000 | -6.81 | -2.71 |
|  | Cryptocurrency/Highly familiar | Cryptocurrency/Less familiar | 4.098^*^ | 0.596 | 0.000 | 2.11 | 6.09 |
|  |  | Money/Less familiar | 3.145^*^ | 0.755 | 0.004 | 0.62 | 5.67 |
|  |  | Recognition/Less familiar | 5.077^*^ | 0.632 | 0.000 | 2.96 | 7.19 |
|  |  | Money/Highly familiar | 0.453 | 0.498 | 0.975 | -1.21 | 2.12 |
|  |  | Recognition/Highly familiar | 0.317 | 0.506 | 0.996 | -1.37 | 2.01 |
|  | Money/Highly familiar | Cryptocurrency/Less familiar | 3.645^*^ | 0.569 | 0.000 | 1.74 | 5.55 |
|  |  | Money/Less familiar | 2.692^*^ | 0.735 | 0.021 | 0.24 | 5.15 |
|  |  | Recognition/Less familiar | 4.624^*^ | 0.607 | 0.000 | 2.59 | 6.65 |
|  |  | Cryptocurrency/Highly familiar | -0.453 | 0.498 | 0.975 | -2.12 | 1.21 |
|  |  | Recognition/Highly familiar | -0.136 | 0.475 | 1.000 | -1.72 | 1.45 |
|  | Recognition/Highly familiar | Cryptocurrency/Less familiar | 3.782^*^ | 0.577 | 0.000 | 1.86 | 5.71 |
|  |  | Money/Less familiar | 2.828^*^ | 0.740 | 0.013 | 0.36 | 5.30 |
|  |  | Recognition/Less familiar | 4.760^*^ | 0.614 | 0.000 | 2.71 | 6.81 |
|  |  | Cryptocurrency/Highly familiar | -0.317 | 0.506 | 0.996 | -2.01 | 1.37 |
|  |  | Money/Highly familiar | 0.136 | 0.475 | 1.000 | -1.45 | 1.72 |
| Efficiency | Cryptocurrency/Less familiar | Money/Less familiar | -1.656 | 0.939 | 0.683 | -4.79 | 1.48 |
|  |  | Recognition/Less familiar | 0.936 | 0.805 | 0.929 | -1.75 | 3.63 |
|  |  | Cryptocurrency/Highly familiar | -3.822^*^ | 0.695 | 0.000 | -6.14 | -1.50 |
|  |  | Money/Highly familiar | -3.238^*^ | 0.665 | 0.000 | -5.46 | -1.02 |
|  |  | Recognition/Highly familiar | -3.230^*^ | 0.673 | 0.000 | -5.48 | -0.98 |
|  | Money/Less familiar | Cryptocurrency/Less familiar | 1.656 | 0.939 | 0.683 | -1.48 | 4.79 |
|  |  | Recognition/Less familiar | 2.592 | 0.971 | 0.213 | -0.65 | 5.83 |
|  |  | Cryptocurrency/Highly familiar | -2.165 | 0.882 | 0.305 | -5.11 | 0.78 |
|  |  | Money/Highly familiar | -1.582 | 0.858 | 0.638 | -4.45 | 1.28 |
|  |  | Recognition/Highly familiar | -1.574 | 0.864 | 0.651 | -4.46 | 1.31 |
|  | Recognition/Less familiar | Cryptocurrency/Less familiar | -0.936 | 0.805 | 0.929 | -3.63 | 1.75 |
|  |  | Money/Less familiar | -2.592 | 0.971 | 0.213 | -5.83 | 0.65 |
|  |  | Cryptocurrency/Highly familiar | -4.757^*^ | 0.738 | 0.000 | -7.22 | -2.29 |
|  |  | Money/Highly familiar | -4.174^*^ | 0.709 | 0.000 | -6.54 | -1.80 |
|  |  | Recognition/Highly familiar | -4.166^*^ | 0.717 | 0.000 | -6.56 | -1.77 |
|  | Cryptocurrency/Highly familiar | Cryptocurrency/Less familiar | 3.822^*^ | 0.695 | 0.000 | 1.50 | 6.14 |
|  |  | Money/Less familiar | 2.165 | 0.882 | 0.305 | -0.78 | 5.11 |
|  |  | Recognition/Less familiar | 4.757^*^ | 0.738 | 0.000 | 2.29 | 7.22 |
|  |  | Money/Highly familiar | 0.584 | 0.581 | 0.962 | -1.36 | 2.53 |
|  |  | Recognition/Highly familiar | 0.591 | 0.591 | 0.962 | -1.38 | 2.57 |
|  | Money/Highly familiar | Cryptocurrency/Less familiar | 3.238^*^ | 0.665 | 0.000 | 1.02 | 5.46 |
|  |  | Money/Less familiar | 1.582 | 0.858 | 0.638 | -1.28 | 4.45 |
|  |  | Recognition/Less familiar | 4.174^*^ | 0.709 | 0.000 | 1.80 | 6.54 |
|  |  | Cryptocurrency/Highly familiar | -0.584 | 0.581 | 0.962 | -2.53 | 1.36 |
|  |  | Recognition/Highly familiar | 0.008 | 0.554 | 1.000 | -1.84 | 1.86 |
|  | Recognition/Highly familiar | Cryptocurrency/Less familiar | 3.230^*^ | 0.673 | 0.000 | 0.98 | 5.48 |
|  |  | Money/Less familiar | 1.574 | 0.864 | 0.651 | -1.31 | 4.46 |
|  |  | Recognition/Less familiar | 4.166^*^ | 0.717 | 0.000 | 1.77 | 6.56 |
|  |  | Cryptocurrency/Highly familiar | -0.591 | 0.591 | 0.962 | -2.57 | 1.38 |
|  |  | Money/Highly familiar | -0.008 | 0.554 | 1.000 | -1.86 | 1.84 |
| *. The mean difference is significant at the 0.05 level. | | |  |  |  |  |  |
